# CimpleG: finding simple CpG methylation signatures

**DOI:** 10.1186/s13059-023-03000-0

**Published:** 2023-07-10

**Authors:** Tiago Maié, Marco Schmidt, Myriam Erz, Wolfgang Wagner, Ivan G. Costa

**Affiliations:** 1 Institute for Computational Genomics, Joint Research Center for Computational Biomedicine, RWTH Aachen University Medical School, Pauwelsstr. 19, Aachen, 52074 NRW Germany; 2 Helmholtz Institute for Biomedical Engineering, RWTH Aachen University, Pauwelsstr. 19, Aachen, 52074 NRW Germany; 3 Institute for Stem Cell Biology, RWTH Aachen University Medical School, Pauwelsstr. 19, Aachen, 52074 NRW Germany

**Keywords:** DNA methylation, Signature selection, Deconvolution, Clinical application

## Abstract

DNA methylation signatures are usually based on multivariate approaches that require hundreds of sites for predictions. Here, we propose a computational framework named CimpleG for the detection of small CpG methylation signatures used for cell-type classification and deconvolution. We show that CimpleG is both time efficient and performs as well as top performing methods for cell-type classification of blood cells and other somatic cells, while basing its prediction on a single DNA methylation site per cell type. Altogether, CimpleG provides a complete computational framework for the delineation of DNAm signatures and cellular deconvolution.

**Supplementary Information:**

The online version contains supplementary material available at 10.1186/s13059-023-03000-0.

## Background

DNA methylation (DNAm) is intrinsically related to chromatin structure and cell differentiation and controls the expression of neighboring genes [1]. The stability of DNAm and the accuracy of genomic methods for quantification of DNA methylation levels makes DNAm a powerful molecular marker for the prediction of age [2–5] and stratification of cancer patients [6, 7]. Furthermore, some loci have a characteristic DNAm profile in specific cells and can therefore be used for cell-type characterization [8, 9] and estimation of cell proportions in tissues via cellular deconvolution [10, 11]. Development of such biomarkers was particularly eased by the rapidly growing number of available DNAm profiles that were measured with Illumina BeadChips, which can address up to 850,000 CpG sites [12]. The epigenetic signatures that are generated with these genomic DNAm profiles often comprise more than 100 CpGs. While the integration of multiple CpGs is considered to increase precision, it greatly hampers application in clinical settings, that require fast, standardized, and cost-effective analysis [13]. To this end, targeted analysis of individual CpGs, e.g., with pyrosequencing, digital droplet PCR, epiTYPER, or amplicon sequencing may be advantageous [14].

A common approach for the detection of DNAm signatures is the use of statistical tests, which characterize differential methylated cytosines (DMC) between samples in two biological conditions [11]. Such approaches are provided by pipelines for analysis of DNAm [15–19] and are mostly based on the use of the limma moderated *t*-test [20] or on fold change statistics [21]. Elastic Net [22, 23] is also commonly used for building DNAm-based models, due to its ability to cope with large dimensional data by the selection of active features during model estimation. For example, a frequently used epigenetic aging clock of Horvath is based on only 346 CpG sites [2] selected with Elastic Net, from DNAm data measured with Illumina BeadChips (27k and 450k). Deep learning approaches displayed high predictive accuracy in cancer prediction, as recently shown in the prediction of primary or metastasis lung cancers [24]. Neural networks, however, work on previously selected DNAm panels with only a small proportion of the initial DNAm sites ($\sim$2000 DNAm sites) and do not indicate the importance of individual DNAm sites.

There are several methods focusing on the problem of reference-based cell deconvolution, i.e., estimating the proportions of cells in a mixture. Jaffe et al. uses *t*-statistics for the selection of the top 100 DNAm sites per cell type (50 hypo- and 50 hyper-methylated). It then explores a nonlinear random effects model, to determine which coefficients are used as predictors for relative proportions of cells. This approach is implemented within the minfi package [25]. The ENmix package [18] is similar to minfi, as it also implements a deconvolution method proposed in Houseman et al. [26], however, it allows for a flexible choice on the number of DNAm sites to be used. IDOL [10, 27] expands on the previous approaches. It first uses a *t*-test to select large cell specific DNAm signatures. These signatures are used to build models on a training mixture data set. IDOL iteratively removes DNAm sites, based on their predictive importance, until an optimal signature for the cell deconvolution problem is found. Finally, EpiDISH performs cell deconvolution with a robust partial correlation based method. It uses for this DNAm signatures selected by a $t-$test, which also overlap with DNAse hypersensitive (or open chromatin) sites [21]. Despite the success of these methods for cell deconvolution and DNAm signature prediction, we are not aware of any computational approach tailored for the selection of small DNAm signatures for either cell type or cell deconvolution problems.

**Fig. 1:**
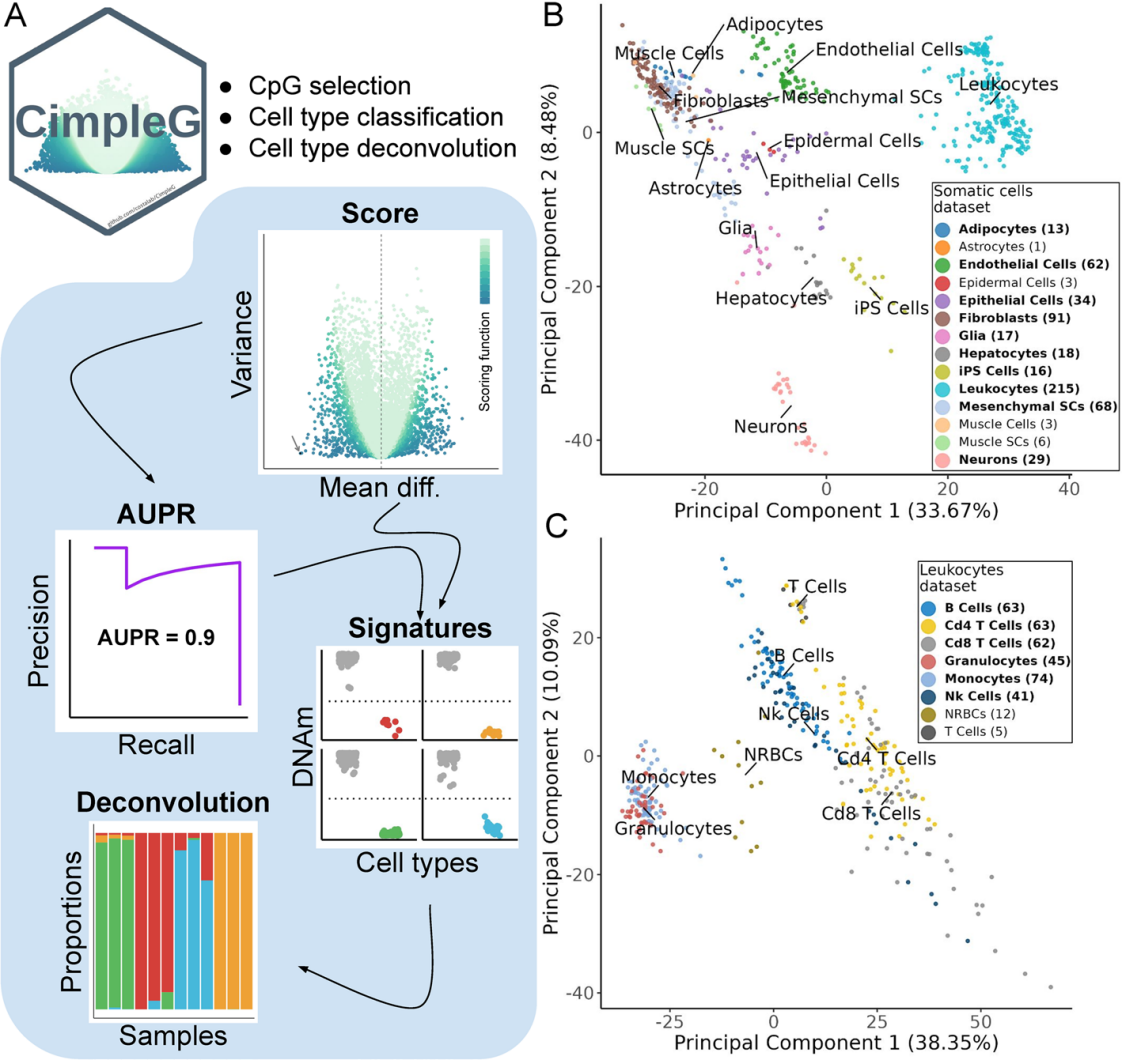
**A** Overview of CimpleG and statistics used for feature selection and downstream applications. **B**–**C** Principal component analysis of the DNAm datasets with somatic cells (**B**) or leukocytes (**C**). Only cell types highlighted in bold, for which we have samples in train ($\ge 10$ positive examples) and test data, were used as target classes. Cell types that are not present in the test data are only used as negative examples (non-target cells)

We have recently described studies on the use of few CpG sites for cell-type deconvolution [13, 28]. Among others, we showed that DNAm signatures with two DNAm sites for fibroblast cells, which we used as a surrogate for fibrosis level, indicated lower survival rates in several cancer types. Building upon this work, we propose and formalize a computational framework named CimpleG for the detection of simple (small) CpG methylation signatures (Fig. 1A). In brief, CimpleG uses a univariate feature selection by combining a *t*-statistic measure with the Area under the Precision-Recall Curve (AUPR) [29] to select the best DNAm sites for cell-type classification. We evaluate CimpleG and competing methods for their performance on cellular prediction on datasets with distinct types of blood cells and other somatic cells for cell-type prediction. Furthermore, we evaluate CimpleG and competing methods in the distinct problem of cell mixture deconvolution on three different cell mixture (real and artificial) datasets.

## Results

### CimpleG framework

CimpleG is a computational framework for the selection of DNAm signatures for cell-type classification (Fig. 1A). It provides a novel feature selection metric to select small DNAm signatures. CimpleG initially uses a *t*-statistic score to pre-select active features followed by the area under the precision-recall curve (AUPR) for feature selection. The use of precision-recall curves instead of the usual ROC curves for feature selection [29] was adopted due to the high imbalance of classes in DNAm cell classification problems, i.e., an average of 15 negatives for a positive example. Next, CimpleG ranks and selects the top candidate CpG sites by combining the score and the AUPR value. These are used to build univariate cell-type-specific classifiers and for cell-deconvolution (Fig. 1A). In addition, the CimpleG framework facilitates the use of alternative feature selection and classification methods (such as random forests [30], Elastic Net [23], and Boosted trees (XGBoost) [31]).

Moreover, CimpleG provides two curated and pre-processed DNAm datasets with a compendium of DNAm arrays with 14 somatic cell types and eight different leukocytes (Fig. 1B–C). These data were pre-processed with well-known state-of-art DNAm-based methods such as SeSAMe [19], minfi [15], and watermelon [32]. The final somatic cells and leukocytes datasets have 576 and 365 samples with 143,291 and 284,706 CpG sites, respectively. We stratified these datasets in train and test samples such that data from the same study are only found as test or training data. Moreover, training and test data were pre-processed independently to avoid leak pre-processing [33] (see Tables 1 and 2 for data characteristics). Principal component analysis of these two datasets shows some separation between major cell types (Fig. 1B–C), while closely related cells (Fibroblast and MSC cells in somatic cell data; CD4 and CD8 T cells in the leukocytes data) can only be discriminated with additional PCs (Additional file 1: Fig. S1).

### Benchmarking the cell-type prediction problem

CimpleG was evaluated in comparison to different alternative methods to generate epigenetic signatures: decision trees, random forests, boosted trees, neural networks, and Elastic Net. We also considered a single-feature DNAm brute force classifier as a baseline. This brute force algorithm evaluates all possible individual markers and ranks these by AUPR. Moreover, we also evaluated CimpleG variants only considering the AUPR (CimpleG AUPR) or the t-statistic (CimpleG Score), which is equivalent to the approach used by state-of-art DNAm analysis and cell deconvolution methods. This was to ensure that the combination of these metrics, was stronger than their individual use. Notably, some models (neural networks, random forests, and decision trees) could not cope with the high dimensional feature size of the datasets. This was mainly due to very large memory usage or days of execution time. Therefore, for these models, as a pre-training step, we have performed an unsupervised feature selection considering variance and co-variance of the DNAm sites [34]. Next, we have used a cross-validation framework to optimize parameters for all methods. We evaluated three main benchmarking metrics, the classification performance as measured by the AUPR, the computational time required and the number of features used by each model. We only build classifiers/signatures for cell types with at least 10 samples in the training data (10 out of 14 in the somatic cells and 6 out of 8 in blood cells), however, we still keep cells with small sample sizes to be used as negative examples (non-target classes). See Additional file 1: Fig. S2 for an overview of the experimental pipeline.

**Fig. 2:**
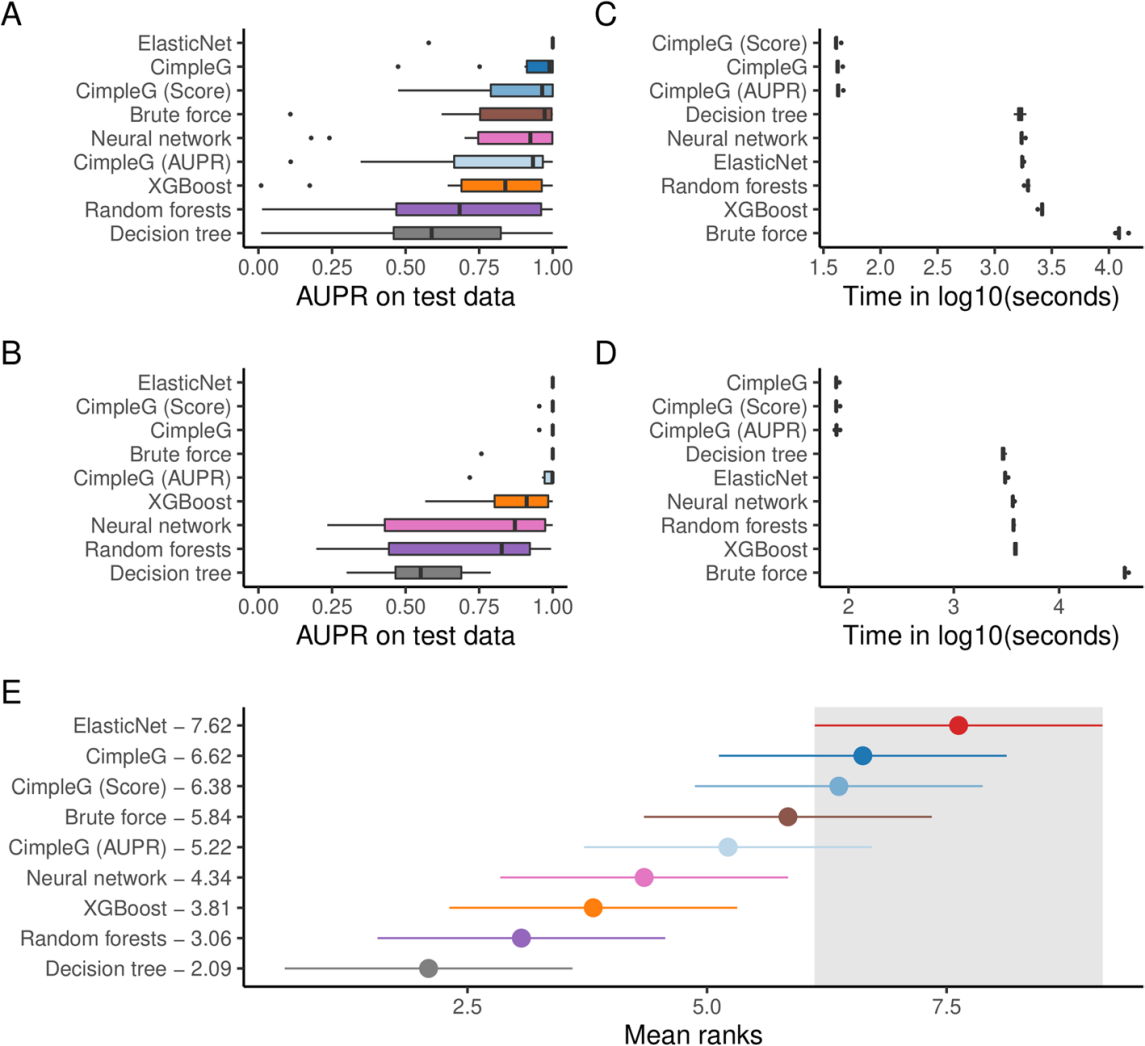
**A**–**B** Classification performance (AUPR) on the test set of the somatic cells (**A**) and leukocytes datasets (**B**). **C**–**D** Computational time required for each method to produce a signature including cross-validation and testing for the somatic cells (**C)** and leukocytes (**D**) datasets. **E** Mean ranks of the methods across all datasets based on the AUPR. The best method, Elastic Net, and its 95% confidence interval (Friedman and Nemenyi post hoc test) is highlighted in gray. Methods that do not overlap at all with the highlighted area, are significantly worse than the highlighted methods

We observed that Elastic Net, CimpleG, and CimpleG (score) have the highest median AUPR for the somatic cells and the leukocyte dataset (Fig. 2A–B), indicating that these are the best performing models. By considering the means of ranks, we observed that Elastic Net, CimpleG, and CimpleG (score) are the three best classifiers in regards to their accuracy on the test datasets. Since the number of target samples differs per target cell type, a relevant question is if there is any association between the number of positive examples (target samples) and the classifier’s accuracy of individual methods (Additional file 1: Fig. S3). We observed that the top-performing methods (Elastic Net and CimpleG) have stable AUPR values across positive sample numbers, which indicates they are robust regarding a low number of positive samples. Regarding computational time, per signature, CimpleG took on average 55.3 s, Elastic Net needed on average 37.6 min, while the Brute force algorithm required on average 6.61 h to generate a signature (Fig. 2C–D). Of note, the computing time of the algorithms presented here does not represent a crucial aspect of the analysis. This is because the computing times are much lower than the time necessary for measuring or pre-processing the DNA methylation data. These results indicate these three methods perform equally well in the DNAm-based cell classification problem, while CimpleG provides a significant speed up for the feature selection problem.

### Selection of DNA methylation sites

**Fig. 3:**
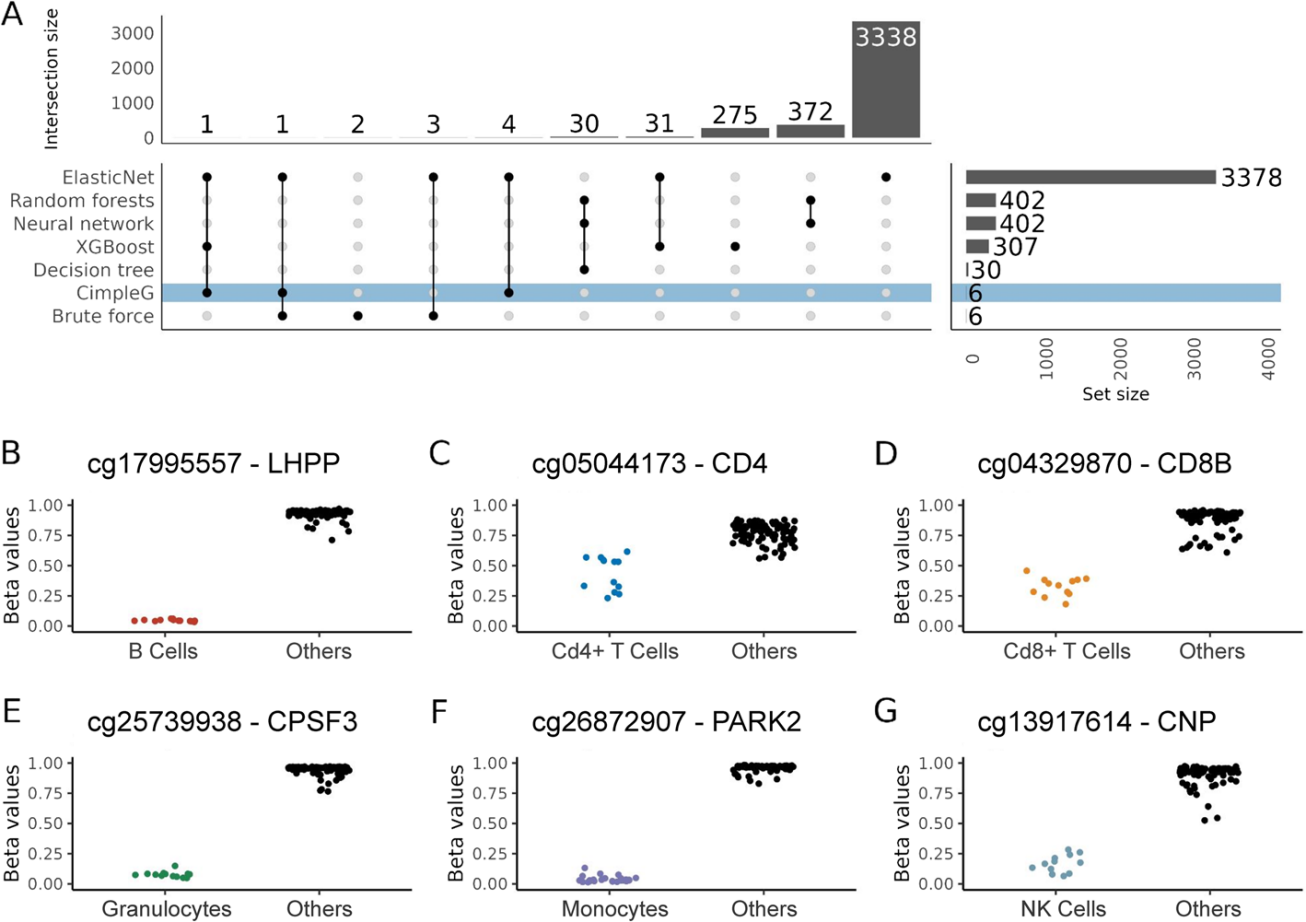
**A** Upset plot showing the total number of selected DNAm sites per method (*y*-axis) and how these are shared per method combinations (*x*-axis) for the leukocytes dataset. Connected dots in a column indicate the combination of methods considered in the *x*-axis. **B**–**G** Beta values of CpG sites selected by CimpleG on the test data. The color of the points corresponds to the target cell type, while points in black correspond to the cell types that are not the targets for that signature

Another relevant point is the number of CpGs that are implemented in the signatures derived by the different methods. Elastic Net selected the largest number of features with 3378 unique features across all six models for the leukocyte cells (Fig. 3A) and 2345 unique features across all ten models for the somatic cells (Additional file 1: Fig. S4A). This is more than all other models combined. The single-feature classifiers (CimpleG and Brute force) selected in total 10 and 6 DNAm sites for somatic and leukocytes data respectively, one CpG per cell type. We observed a high overlap of these features with the ones selected by the Elastic Net, i.e., all 16 DNAm selected by CimpleG were also part of the DNAm sites selected by Elastic Net. Interestingly, features selected by random forests, neural networks or decision trees were quite distinct from other methods. This rose from the fact that these methods require the previous use of univariate filters, due to their incapacity to deal with large dimensional inputs. Altogether these results show the ability of CimpleG in the delineation of small DNAm signatures.

Furthermore, it is interesting to look at the specific signatures generated by CimpleG (Fig. 3B–G; Additional file 1: Fig. S4B–K) as these genomic locations could provide biological insight into the cells themselves (see Additional file 2: Table S1 for complete results). Some DNAm sites are close to genes functionally related to cells, i.e., DNAm sites close to genes for CD4 (cg05044173, Fig. 3C) and CD8 (cg04329870, Fig. 3D) are selected as markers for CD4+ and CD8+ T cells. A DNAm site (cg01537765, Additional file 1: Fig. S4B) in the body of LIPE (Lipase E, Hormone Sensitive Type) is selected as a marker for adipose cells. This gene is known to function in adipose tissue by hydrolysing stored triglycerides to free fatty acids [35]. Finally, a CpG (cg10624122, Additional file 1: Fig. S4J) in the promoter of the epithelial mesenchymal transition related transcription factor, TWIST1 [36], is selected as a marker for mesenchymal stem cells. Moreover, other CpGs are close to genes with cell-specific expression patterns according to the human protein atlas [37]. An example is the CpG cg10673833 (Additional file 1: Fig. S4I) close to the gene MYO1G, which is a good marker for lymphocytes; and the CpG cg23882131 (Additional file 1: Fig. S4E) close to MRGPRF, which is a marker for fibroblasts. While the functional association of other markers is not evident, it needs to be considered that DNAm status does not generally translate directly to the expression of neighboring genes.

Of note, some of the samples used in the leukocyte data were derived from cord blood or non-healthy samples. One important question is if the ontogenetic differences in hematopoiesis or specific diseases might affect the selected signatures. As observed in the Additional file 1: Fig. S5, in our analysis, none of covariates impact the DNAm values of the signatures derived by CimpleG.

### Benchmarking the cell-type deconvolution problem

Next, we evaluate DNAm signatures and model predictions on a cell deconvolution problem in leukocytes. For this, we use either the DNAm sites (for models with small signatures; CimpleG, Brute Force) or the model prediction scores (for Elastic Net, Random Forests, Boosted Trees, and Neural Networks) to build reference matrices for each model vs. cell type. We used these as input for the deconvolution method, the non-negative least squares (NNLS) algorithm [38], which is a common deconvolution method for DNAm due to its simplicity and being among the top performers in a recent benchmarking study [39]. We inspect the performance of state-of-art deconvolution methods and frameworks (IDOL [27]; EpiDISH [21]; ENmix [18]; and minfi [15]). Methods were trained on an expanded leukocyte reference dataset with 56 samples comprising 12 different cell types when possible. This is in contrast to the dataset that we have compiled for the classification problem, which had many more samples but covered only six major leukocyte cell types. Next, all methods were evaluated based on predictions on two artificial blood sample mixtures (Leukocyte datasets 1 and 2) and a real mixture data set (Leukocyte dataset 3) that were recently reported by Salas et al. [27]. The Leukocyte 1 and 2 datasets are based on mixtures of twelve different cell types. Some of the deconvolution methods (EpiDISH, ENmix, and minfi) are based on pre-trained signatures/reference datasets with only six or seven major leukocytes. We therefore simplified the cell annotation of Leukocyte 1 and 2 data sets by combining their annotation towards six major cell types for evaluation of these approaches. A similar procedure was performed in the Leukocyte data set 3, which only has mixture values for five major lymphocyte populations (see the “Methods” section for details). For all methods where it is possible to control the number of CpG sites (CimpleG, IDOL, and ENmix), we evaluate them with either a large signature (default parameters and 10-CpG sites per cell for CimpleG) or small signatures (1–2 sites per cell)^1^.

**Fig. 4:**
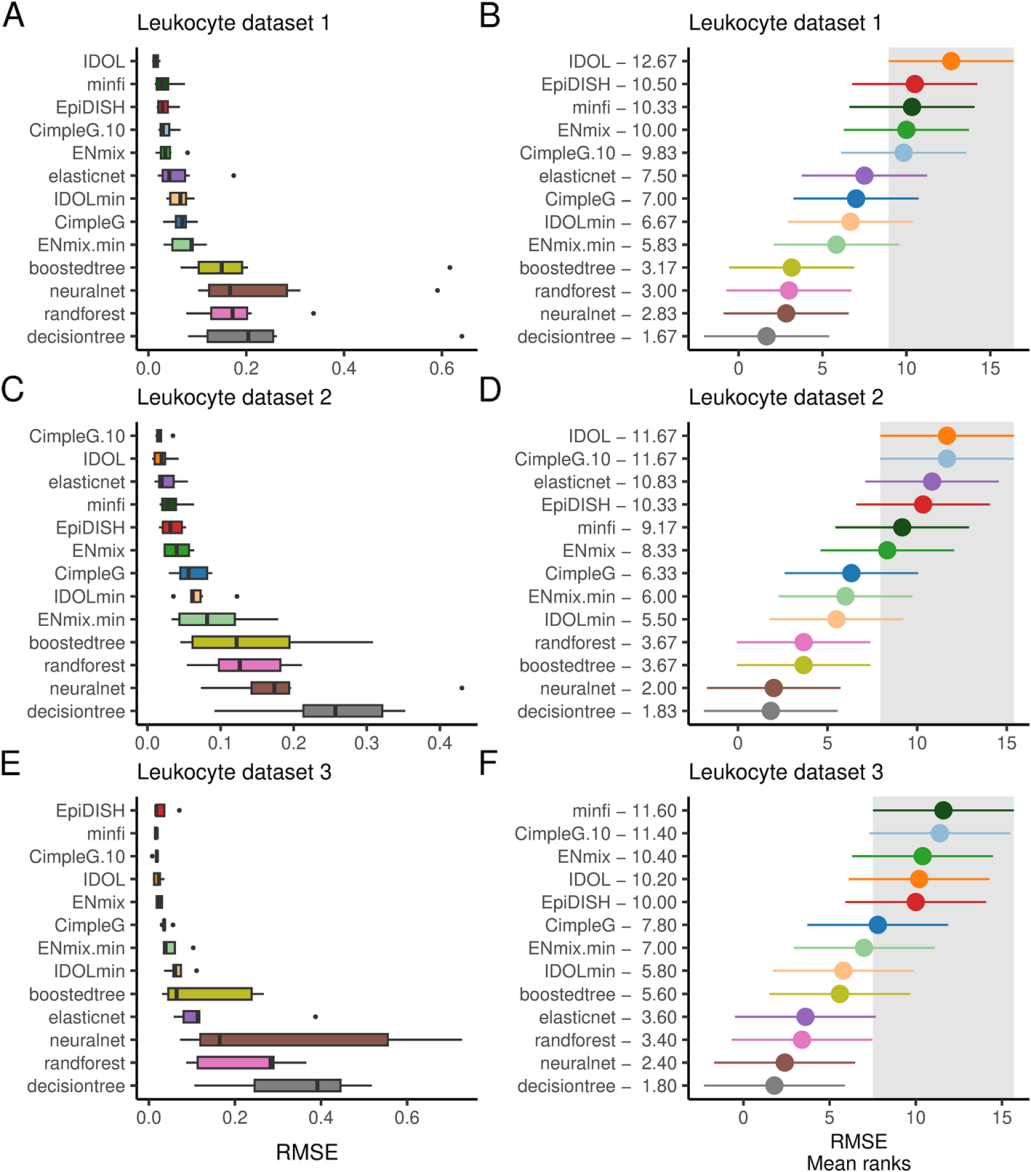
RMSE (left) and its mean ranks (right) are shown for each classifier/deconvolution method and for the Leukocyte 1 (**A**–**B**), 2 (**C**–**D**), and 3 (**E**–**F**) data sets. Methods are ranking from top-down regarding the best performance (lowest RMSE). For the RMSE mean rank plots, the best method, and its 95% confidence interval (Friedman and Nemenyi post hoc test) is highlighted in gray. Methods whose average RMSE does not overlap at all with the highlighted area, are significantly worse than the top performing method. CimpleG.10 is based on 10 CpGs per cell type, while ENMix.min and IDOLmin are versions of IDOL and ENMix using the lowest possible number of CpGs

Considering the problem of deconvolution of major (5–6) leukocyte cells, we observe that IDOL had highest ranking when considering the lowest prediction error (lowest RMSE). IDOL was first in 2 out of the 3 data sets followed by CimpleG with 10 CpGs (second in 2 data sets) and minfi (one time first and another time third) (Fig. 4). If we only consider methods based on small signatures (CimpleG, IDOLmin, ENmix.min), we observe that generally, CimpleG has a lower RMSE than the competing methods. On average across these datasets, CimpleG had a mean RMSE of 0.0561, while the top three methods, IDOL, CimpleG.10, and minfi, had a mean RMSE of 0.0188, 0.0240, and 0.0281, respectively, using much larger signatures. IDOLmin and ENmix.min had a mean RMSE of 0.0668 and 0.0743, respectively. Similar results were observed on rankings based on $R^2$ statistics (Additional file 1: Fig. S6–S7).

We also evaluated a selection of methods on the two artificial mixture data sets (Leukocytes 1 and 2). Here, the deconvolution problem is on the more granular annotation, with 12 different leukocyte cell types (Additional file 1: Fig. S8). IDOL obtained the lowest RMSE (with an average of 0.0199) followed by CimpleG with 10 CpGs (with an average of 0.0253). As before, CimpleG with a single CpG per cell type, obtained the lowest average RMSE when comparing with other small signature methods. CimpleG had an average RMSE of 0.0549 vs IDOLmins’ 0.0633.

A crucial aspect is the number of DNAm sites used for each methods. CimpleG only required 12 sites, while IDOLmin, ENmix.min, minfi, ENmix, CimpleG.10, EpiDISH, and IDOL required, respectively, 13, 24, 100, 100, 120, 333, and 1200 DNAm sites. Therefore, CimpleG requires at least 10 fold less DNAm sites than top performing methods IDOL, CimpleG.10 and minfi. Altogether, we observe that CimpleG is a competitive approach for cell deconvolution when based on 10 CpG sites per cell type, while being the best performing method for small signatures.

## Discussion

Despite a huge number of DNAm biomarkers based on large CpG signatures, hardly any of them have been translated to clinical practice [14]. Many of the epigenetic signatures comprise a multitude of CpGs, which requires microarray or deep-sequencing methods that are difficult and expensive to implement for routine applications. We propose here CimpleG, which explores both t-statistic and AUPR scores, to select a single DNAm site per cell type of interest. In the context of cell signature selection, the performance of CimpleG was contrasted to other state-of-art methods, such as t-statistic, Elastic Net, Random Forests, Boosted Trees, and Neural Networks. Our results indicate that Elastic Net, which is broadly used for aging signatures [2], is the overall best method, but tends to select relatively large signatures that can comprise thousands of DNAm sites for its predictions. CimpleG performed as accurately as Elastic Net while focusing on only one DNAm site per cell type. An important, but poorly explored aspect, is the effect of biological variability on the cell signature and deconvolution methods. It is unclear how age, health status (i.e., infections or physiological diseases) might influence the cell specific signatures. While we observe low effects of some covariates, such as disease vs. healthy and age (new born vs. adult) in our data set, larger DNA methylation data with rich clinical and sample annotation are required for an in depth analysis.

Another major scientific contribution of this work is the comparative analysis of CimpleG and state-of-art cellular deconvolution methods. We evaluated 13 approaches including four state-of-art cell deconvolution frameworks in two artificial mixtures and 1 real mixture data set for classification of major (5–6 cell types) or specific (12 cell types) leukocyte cells. Our results confirm the previously reported superior performance of the IDOL algorithm [27]. IDOL is the only deconvolution method performing DNAm signature selection in two steps, it first delineates cell-type-specific features from DNAm with isolated cells. This signature is then refined using a training mixture data set. Nevertheless, CimpleG obtained competitive results when selecting 10 CpGs per cell type. More importantly, CimpleG obtained the overall smallest error (RMSE) in all evaluated data sets, when compared to IDOLmin and ENmix.min. Moreover, we observe that these results are equivalent for data that is either based on artificial (leukocyte datasets 1 and 2) or real mixtures (leukocyte dataset 3), despite the fact that CimpleG’s signatures were 100x smaller than standard deconvolution methods.

While the deconvolution of hematopoietic subsets is just one example for epigenetic signatures, it also addresses a relevant clinical need. Current flow cytometric measurements are only applicable for fresh blood samples and do not facilitate analysis of dried blood spots, coagulated material, or retrospective analysis. Furthermore, it is not trivial to standardize flow cytometry settings. These clinical needs can be met by epigenetic deconvolution of leukocyte subsets [28, 40, 41]. However, for clinical translation, it is crucial to have very robust and standardized measures that are applicable over decades. The entire process, including data analysis, has to be accredited for clinical application, and this is usually hampered for genome wide analysis with changing analytical platforms [14]. In principle, epigenetic diagnostics with genome-wide analysis is feasible - deep sequencing technology is also used for screening of mutations and chromosomal abnormalities in the clinics - however, the accreditation process is more challenging, as compared to targeted assays. For example, the Illumina BeadChip microarray platforms are so far not accredited for clinical diagnostics. The very small epigenetic signatures derived by CimpleG are thus a big advantage for clinical application, as they facilitate development of analytical procedures with targeted assays for specific genomic regions, e.g., by pyrosequencing, ddPCR, or amplicon sequencing. In fact, we have recently used CimpleG to identify cell-type-specific CpGs for various hematopoietic subsets that could be validated by ddPCR in fresh blood samples and dried capillary blood from finger pricks of 150 patients (Hubens et al., manuscript currently under revision).

Furthermore, it is crucial to consider the right metrics, when evaluating cell deconvolution methods for predictive performance. Common metrics include the root mean squared error (RMSE) and the *R*-squared ($R^2$) [10, 25, 27]. We have noticed during our analysis that the commonly used $R^2$, which evaluates how well the predicted cell proportions fit to the model, might be associated with a large RMSE (Additional file 1: Fig. S9–S10). In this context, the problem arises when the estimated model systematically over-estimates (or under-estimates) the predicted proportions. An alternative for this is the diagonal $R^2$, which evaluates the error of the fit to the diagonal line where true predictions should lie. As shown in our example (see Additional file 1: Fig. S11), we observe that the values from the commonly used $R^2$ definition (denoted $R^2(fit)$ here) can be small despite large RMSE and systematic over-estimation of cell proportions. A similar issue has been previously discussed on statistical literature [42, 43]. In our view, the $R^2$ (in all its different forms) should not be used for evaluation of cell deconvolution, whereas the RMSE is a much more appropriate metric.

Altogether, the CimpleG framework can be seen as a general pipeline for DNAm signature selection. It is implemented in R and provides functionalities to estimate both small DNAm signatures (using CimpleG), but also to derive Elastic Net and other machine learning methods evaluated here. It provides two large, manually curated and normalized datasets for the testing and benchmarking of methods. Moreover, cell-type classifiers can be integrated with the NNLS method for cellular deconvolution, which makes it the first end-to-end pipeline for the estimation of cell-specific small DNAm signatures to cellular deconvolution. We are not aware of any other computational package addressing these.

There are further aspects to be explored in the future. One technical problem usually encountered in array based DNA methylation data sets is missing values, which usually increase with data set size. Currently, none of the evaluated deconvolution workflows, except for CimpleG, supports predictions with missing values via imputation [44]. Further problems are the impact of missing reference cell types or the use of CimpleG signatures for cell-of-origin detection from circulating cell free DNA [45]. From a technical perspective, there is a need for data structures for the efficient handling of ever-increasing DNAm datasets. Another relevant issue are approaches for automatic integration with data repositories such as Gene Expression Omnibus. Note, however, that the lack of a consistent cell-type annotation, i.e., as provided in cell ontology [46], on such repositories, makes manual cell-type annotation still a requirement.

## Conclusion

We propose a computational framework named CimpleG for the detection of small CpG methylation signatures used for cell-type classification and deconvolution. Small signatures are important due to their potential application in clinics. Our extensive benchmarking for both cell-type detection and cell deconvolution indicates that CimpleG is fast and can perform as well as state-of-the-art approaches, which require large DNA methylation signatures. This works also provide two large benchmarking data sets with either leukocytes and epithelial cells, which represents a useful benchmarking resource for future approaches. Taken together, CimpleG provides a valuable tool for clinician scientists as an easy-to-use stand-alone framework for the identification of CpG sites that are best suited as a biomarker for targeted DNAm analysis.

## Methods

### CimpleG for signature selection

CimpleG receives as input a matrix $\textbf{X} \in (0, 1) \subset \mathbb {R}^{p\times n}$, where $x_{ij}$ represents the DNAm values for site *i* in observation *j*, *n* is the number of observations and *p* is the number of DNAm sites. This essentially describes a matrix of beta-values. The beta-value is the ratio of methylated probe intensity versus the overall intensity in the Illumina methylation assays  [47, 48]. Alternatively, CimpleG can also, receive as input a matrix of *M*-values, these are the log2 ratio of intensities of methylated versus unmethylated probes [47], a metric widely used in microarray assays and that shows some statistical advantages specifically when looking at the low and high end of the methylation range [47]. CimpleG also receives a vector $y \in \{0,1\}^p$, which is 1 if observation *j* is of a cell type of interest and zero otherwise (others).

CimpleG characterizes a DNAm site *i*, for the cell type of interest, by the difference in means (Eq. 1) and the sum of variances (Eq. 2).

1 $$\begin{aligned} \Delta _{i}^{cell} = \mu _i^{cell} - \mu _i^{others} \end{aligned}$$

2 $$\begin{aligned} \sigma _{i}^{cell} = \textrm{var}_i^{cell} + \textrm{var}_i^{others} \end{aligned}$$

where $\mu _{i}^{cell} = \frac{\sum _{j} \textbf{1}(y_{j}=1)x_{i j}}{|\textbf{1}(y_{j}=1)|}, \mu _{i}^{other} = \frac{\sum _{j} \textbf{1}(y_{j}=0)x_{i j}}{|\textbf{1}(y_{j}=0)|}$, and 1 is an indicator function. Similarly, $\textrm{var}_i^{cell}$ and $\textrm{var}_i^{others}$ provides variance estimates for DNAm site *i*.

CimpleG first selects active features such that $\Delta _i$ is high and $\sigma _i$ is low. For this, it ranks DNAm sites according to *a* with

3 $$\begin{aligned} a_{i}^{cell}=\frac{\sigma ^{cell}_i}{(\Delta ^{cell}_i)^{b}} \end{aligned}$$

where *b* is a non-negative even constant integer that determines how much importance features with high difference in means should have. The larger *b* is, the larger the bias will be towards selecting DNAm sites with a higher difference in means $\Delta$, regardless of the sum of variances $\sigma$. Of note, for $b=1$, Eq. 3 is equivalent to a *t*-student statistic for the comparison of mean values of two groups. We set *b* to 2 as default. Finally, a quantile function *Q*(*p*) is used to generate a threshold below which sites with a value of *a* are selected. By default, we select $0.5\%$ sites as active features.

CimpleG performs a balanced stratified K-fold cross-validation loop on the training data and uses the previous procedure to find the *f* active features for each fold. CimpleG sets $k=10$ as a default number of folds, unless the number of target class samples $n<k$, in which case $k=n$. To evaluate individual features as one-feature classifiers, it uses an area under the precision-recall curve (AUPR) procedure. This methodology is inspired by a feature selection method based on the area under the curve (AUC) proposed by Chen and Wasikowski [29]. In short, we evaluate the precision and recall for a linear classifier $x_i$ in regards to *y*. CimpleG adopts an AUPR curve instead of AUC as an accuracy metric, as it makes the procedure less affected by class imbalance [49].

After evaluation of all folds, we compute a final metric by considering DNAm sites *i*, which minimizes the score $a_i$, while maximizing the average AUPR values in training and validation ($AUPR_{ti}$ and $AUPR_{vi}$, respectively) and being active features ($f_i$) in most folds, that is:

4 $$\begin{aligned} C^{cell}_i = \frac{(\alpha _1 * a^{cell}_i + \alpha _2 * \textrm{AUPR}^{cell}_{ti} + \alpha _2 * \textrm{AUPR}^{cell}_{vi} )}{f_i} \end{aligned}$$

where *i* denotes a given feature. Finally, $\alpha _1$ and $\alpha _2$ are constants by which we control how strong the influence of *a* and the *AUPR* values will have on the final score. We set these as 0.8 and 0.2, respectively. We then use this score to rank all active features for a given cell type.

### Data for the cell-type classification problem

We have compiled and curated two human cell-type DNA methylation datasets that were measured with Illumina Infinium Human Methylation 450k and EPIC BeadChip arrays and deposited in the Gene Expression Omnibus (GEO) [50, 51]. Cell-type information and other metadata were also obtained from GEO. Note that although some samples are shared between the somatic cells and leukocyte datasets, these were treated as independent datasets and therefore the samples were processed and treated as part of their respective dataset.

#### Somatic cells

We have searched for DNAm array data with an emphasis on purified, well-characterized, non-malignant cells in GEO. After manual curation, we had 576 samples, spanning over 14 different cell types, from 46 distinct studies (see Table 1 for overall statistics) [13]. We provide a detailed sample sheet in Additional file 2: Table S2, which includes GEO sample IDs (GSMs), GEO series/studies IDs (GSEs), cell type, assigned dataset (train/test) and relevant covariates.

**Table 1:** Somatic cells data summary

	Train dataset	Test dataset	*Total*
**Adipocytes**	10 (2.4%)	3 (1.8%)	13 (2.3%)
Astrocytes	1 (0.2%)	0 (0%)	1 (0.2%)
**Endothelial cells**	32 (7.8%)	30 (18%)	62 (11%)
Epidermal cells	3 (0.7%)	0 (0%)	3 (0.5%)
**Epithelial cells**	16 (3.9%)	18 (11%)	34 (5.9%)
**Fibroblasts**	52 (13%)	39 (24%)	91 (16%)
**Glia**	10 (2.4%)	7 (4.2%)	17 (3.0%)
**Hepatocytes**	15 (3.6%)	3 (1.8%)	18 (3.1%)
**iPS cells**	13 (3.2%)	3 (1.8%)	16 (2.8%)
**Leukocytes**	182 (44%)	33 (20%)	215 (37%)
**Mesenchymal SCs**	56 (14%)	12 (7.3%)	68 (12%)
Muscle cells	3 (0.7%)	0 (0%)	3 (0.5%)
Muscle SCs	0 (0%)	6 (3.6%)	6 (1.0%)
**Neurons**	18 (4.4%)	11 (6.7%)	29 (5.0%)
*Total*	411 (100%)	165 (100%)	576 (100%)

The highlighted rows indicate the cell types that were used to train the classifier.

The other cell types were only part of the training or test dataset for additional control

#### Leukocyte cells

As with the previous dataset, we have searched for DNAm array data in GEO; however, here, we focused on purified leukocytes. After manual curation, we had 365 samples, for eight different cell types from 12 different studies (see Table 2 for overall statistics). We provide a detailed sample sheet in Additional file 2: Table S3, which includes GEO sample IDs (GSMs), GEO series/studies IDs (GSEs), cell-type, train/test dataset assignment, and relevant covariates.

**Table 2:** Leukocyte data summary

	Train dataset	Test dataset	*Total*
**B cells**	50 (19%)	13 (12%)	63 (17%)
**CD4 T cells**	50 (19%)	13 (12%)	63 (17%)
**CD8 T cells**	50 (19%)	12 (12%)	62 (17%)
**Granulocytes**	32 (12%)	13 (12%)	45 (12%)
**Monocytes**	50 (19%)	24 (23%)	74 (20%)
**Nk cells**	29 (11%)	12 (12%)	41 (11%)
NRBCs	0 (0%)	12 (12%)	12 (3.3%)
T cells	0 (0%)	5 (4.8%)	5 (1.4%)
*Total*	261 (100%)	104 (100%)	365 (100%)

The highlighted rows indicate the cell types that were used to train the classifier.

The other cell types were only part of the training or test dataset for additional control.

*NRBCs* nucleated red blood cells

#### DNA methylation data and quality control

Both datasets were independently pre-processed. Raw data (.IDAT files) was downloaded from GEO. If .IDAT files were not available, the tabular data with probe intensities was used. For datasets where IDAT files were available, the SeSAMe pipeline (sesame R package v1.16.1 [19]) was used setting the pre-processing code argument to “QGCDPB.” Briefly, this includes, probe filtering regarding low mapping quality, single sample normalization (ssNoob) [15], nonlinear dye-bias correction and p-value with out-of-band array hybridisation masking/filtering. For samples where only intensity matrices were available, beta mixture quantile normalization (BMIQ, wateRmelon R package v1.34.0 [32]) for type II bias correction was used. After merging the data obtained from the .IDAT files and the tabular data with probe intensities, we remove samples for which over half of the probes are missing, followed by eliminating all probes that still have missing data. Furthermore, we only considered CpGs that were provided by the 450K and EPIC BeadChip platforms. With this procedure, the final datasets have 143,291 and 284,706 features for the somatic and leukocyte cells, respectively. This process filters out a large number of probes, however ensures that the probes kept have a higher mapping quality and should generalize better in unseen datasets.

### Additional DNAm methods for classification

CimpleG also allows the user to train classifiers using a number of different machine-learning algorithms to generate complex methylation signatures. For this, we explore methods exposed by the tidymodels framework [34] and provide a wrapper around five popular models with different levels of complexity (Elastic Net, Decision Trees, Random Forests, Boosted Trees, and Neural Networks). Each method has a set of hyper-parameters that need to be tuned in order to produce proper results. We chose a sensible set (in regards to computational resources) of hyper-parameters to be tuned via grid-search in a stratified cross-validation (CV) loop. After the CV loop, we fit the models on the whole training dataset with the previously tuned hyper-parameters. Due to the fact that some methods did not cope with the original feature size (Decision Trees, Random Forests, and Neural Networks) of DNA methylation data (>100,000 features), we had to apply a filter as a pre-training step in order to remove features that are highly sparse, features that show linear combinations between them and features that have large absolute correlations with other variables. These filters are applied as a pre-training step. The implementation of these methods is out of the scope of this paper but we describe them briefly here.

#### Elastic Net

Elastic Net [22] is a regularized linear regression method that is able to perform feature selection in arbitrarily large datasets. It can also be used for two-class classification problems within a logistic regression framework. Elastic Net has a regularization parameter $\lambda$ that controls penalization, where higher values indicate higher penalisation (lower number of features). Additionally, the mixture parameter $\alpha$ controls the type of regularization, where if $\alpha = 0$ we have a pure ridge regression model, and if $\alpha = 1$ we have a lasso regression model. Here, we optimize both $\alpha$ and $\lambda$ during cross-validation. We used the glmnet package [23] as the underlying engine for these models.

#### Decision Trees

Decision Trees are simple models that are defined by a set of if/else statements creating a tree-based structure. These models are very easy to understand, but they tend to be inaccurate and unstable and also have several hyper-parameters to tune. We used the rpart package[52] as the underlying engine for these models. Here, we consider the maximum tree-depth as the single parameter to be tuned during the CV loop, while most other parameters are set as default values. The exceptions are the engine-specific parameters “maxsurrogate” and “maxcompete” which were set to 2 and 1, respectively, in order to reduce execution times.

#### Random Forests

Random Forests is an ensemble based method that builds predictions by combining decision trees in a bagging fashion. In essence, random forests create a large ensemble of independent decision trees, which are based on a random and small selection of features, and then builds its predictions using a combination of the predictions of all the individual decision trees. We used the package ranger [53] as the underlying engine for these models. Here, we set the number of independent decision trees as the parameters to be tuned during the CV loop, while other parameters, except for “oob.error,” “respect.unordered.factors,” and “importance,” were set to default. The aforementioned engine-specific parameters were set to “FALSE”, “order,” and “impurity_corrected,” respectively. This was in order to save on computation time, to better fit our experimental design (2-class classification problem) and to be able to measure feature importance after model training.

#### Boosted Trees

Boosted Trees are also models that are built on ensembles of decision trees. While boosted trees also typically create a large number of decision trees, the selection of samples and features are based on the predictive performance of the previously trained decision trees. To perform predictions all the decision trees in the ensemble are combined to produce a result. Here, we used the package xgboost [54] as the underlying engine for boosted trees. Similarly to the random forests models, we set the number of trees in the ensemble as the parameter to be tuned during the CV loop. To improve computation times and to better fit our experimental design, we set the parameter “mtry” to 100. Furthermore, we set the engine-specific parameters “objective” to “binary:logistic,” “eval_metric” to “aucpr,” and finally, “maximize” to “TRUE”. Other parameters were kept as default as per the tidymodels interface.

#### Neural Networks

Neural Networks are models that work with the concept of combining layers of interconnected computational neurons (perceptrons) to produce a prediction. Here we focus on a simple, feed-forward neural network with three layers, the input layer, a single hidden layer, and the output layer. We used the nnet package [55] as the underlying engine for our networks. In our wrapper implementation, we set the number of hidden units (perceptrons in the hidden layer) and the complexity penalty parameter as the parameters to be tuned during the CV loop. To have a sensible computational footprint, we kept the epochs (number of training iterations for each network) to 100, as default. However, due to the nature of our data (high-dimensionality, even after filtering), we had to set the engine-specific parameter “MaxNWts,” which controls the maximum number of weights in the model, to one million, otherwise, the models would not fit. This parameter is necessary but makes the fitting process more computationally expensive.

### Experimental design

The overall design is shown in Additional file 1: Fig. S2. First, we divided the data into a train and a test set. The train set was used to train and fit the models within a cross-validation setup while the test set was used as an independent assessment of the models after training concluded. The split in the data was manually curated. To avoid bias, we had to ensure that (1) samples from the same study were exclusively in the train set or the test set and (2) whenever possible given (1), the proportion of samples for a given cell type across the different cell types was kept stable between the train and test sets so that these were as balanced as possible. Normalization was performed independently for the train and test data as previously described. Since some algorithms (neural networks, decision trees and random forests) cannot cope with the high dimensionality of the data, CimpleG enforces, during training but before the cross-validation step, a correlation, a co-linearity and a variance-based filter. Therefore, for these three algorithms, the universe of features is reduced to 360 and 402 CpGs for the somatic and leukocytes datasets respectively.

Next, we performed model training for every evaluated approach and for every relevant cell type within the somatic cells and leukocyte datasets (Tables 1 and 2, respectively). Note that, each model is trained independently for each different target class. This means that, for example, we will have six different and trained random forest models for the leukocytes dataset (that has 6 different target classes). For model selection and parameter optimization, we performed stratified cross-validation (10-fold) in the training dataset. The same folds were used for all evaluated models and cell types. See the text above for a description of the optimized parameters. Finally, we assess the performance of each method using the test dataset. The classification performance of each method is evaluated using the Area under the Precision-Recall curve. Other metrics considered for the evaluation and comparison of the models were the time of execution (which includes model optimization) and the number of selected features (if supported by the method). To quantitatively understand how each model ranks against the other, we computed the overall mean ranks (based on the AUPR) as well as the Friedman-Nemenyi post hoc tests. With these, we can assess if different methods perform significantly better than others (Fig. 2).

### CimpleG for cell deconvolution

Let *V* be a matrix with DNAm levels of mixture samples, *W* is the cell-type specific methylation values (our reference matrix) and *H* would be the unknown cell-type proportions per sample. Cell deconvolution can be posed as a matrix factorization problem, that is:

5 $$\begin{aligned} V = W \cdot H. \end{aligned}$$

Given that all these matrices are positive, this problem can be solved with a non-negative least square algorithm (NNLS) [39].

For our signatures, we generate *W* in two distinct ways depending on the type of model the signatures originated from. If the signatures were generated from one of the simple methods (CimpleG or brute-force), then we take their DNAm site selection and build a reference matrix using the average DNAm values of each CpG per class label. Otherwise, for each machine learning method, we build the reference matrix by using the average classification score per classifier, per cell type. To perform cellular deconvolution on the matrix *V*, we again use two different approaches. For the simple methods, we take the methylation level of the DNAm signature sites. However, for the complex methods, we use the classification score of each classifier for the different samples. Taken together, the *W* and *V* matrices are fed to the NNLS algorithm to reconstruct *H*.

For all methods within CimpleG, in the likely scenario that the data we are trying to deconvolve includes missing values, CimpleG will perform mean-imputation so that deconvolution can take place and it will warn the user of how many probes were missing and if applying this procedure makes sense for their data. Although the main focus of CimpleG is on the of use small signatures, before training, the user can also choose to perform deconvolution using the default CimpleG procedure but with a higher number of probes (i.e., CimpleG.10 in Fig. 4). Furthermore, CimpleG also allows the user to use other deconvolution algorithms such as non-negative matrix factorization (NMF) [56], robust partial correlations (RPC) [21], Cibersort [57] and constrained projection (CP) [26]. The last three methods are supported in the back-end by the EpiDISH package [21].

### Competing methods for cell deconvolution

In order to assess how the deconvolution performed by CimpleG (and the methods included therein) compares to other state-of-the-art methods, we selected a number of methods widely used for reference-based deconvolution of DNAm data. These were IDOL [10, 27], EpiDISH [21], minfi [15, 25], and ENmix [18]. Furthermore, whenever possible, we gauge how, for the same method, having signatures of different sizes (CimpleG, IDOL, and ENmix) affects its performance. Of note, most methods (EpiDISH, minfi, and ENmix) provided their own CpG signatures. This is not the case for CimpleG and IDOL, which both explore a 12 cell-type reference data set (see below) for DNAm signature delineation.

#### IDOL

IDOL (Identifying Optimal Leukocyte-differentially methylated region Libraries, v0.0.0.9000) [10, 27] is a method specifically targeting the problem of cell mixture deconvolution. It can be used both as a standalone workflow, capable of identifying and using a panel of probes for deconvolution, as well as using a pre-generated reference signature. In this study we focus on the former, as most methods (see below) already cover the latter.

IDOL’s training process consists of two different steps. First using its function *CandidateDMRFinder.v2*, it selects a panel of candidate probes. This selection is based on *t*-tests run across the contrasts, in this case cell-types, in the data. The dataset used here was the Purified 12 leukocytes data (see below) with 56 different cell-specific samples, covering 12 different cell-types. Under our experimental setup, this step reduces the number of probes available for consideration from 689,105 down to 3540. Then it uses its iterative approach, with the *IDOLoptimize* function by exploring a training mixture data set to optimize the selection of probes to solve the cell mixture deconvolution problem. This process is guided through the use of the RMSE and R²(fit) metrics to determine if a given subset of probes, provides a better result than the previous iteration. We use the same mixture data set (6 samples of artificial mixtures) as used in [27]. Finally, with the selection finished, deconvolution is run with the *projectWBCnew* function. The deconvolution method itself being based on the often called Constrained Projection (CP) algorithm [26].

As mentioned above, for the refining of DNAm signatures, we use the same mixture training dataset as in [27]. Six samples, artificially mixed, composed of 12 different cell types. These were samples with the accession numbers GSM5121366, GSM5121364, GSM5121357, GSM5121367, GSM5121362, and GSM5121365. The pre-processing procedure applied to these data was the same as the one used for the Purified 12 leukocytes data (see below).

We should note that the *IDOLoptimize* step can take a rather long amount of time, with IDOL taking exactly 2 h to execute, while IDOLmin took a little over 9 min to execute. This was achieved while running in parallel on a 6-core setup, with otherwise default parameters, except for the libSize parameter which was set to 13 for IDOLmin (12 cell-types + 1, as this was the minimum that allowed it to run) and 1200 for IDOL to reflect the optimal procedure in described in its most recent study [27]. For comparison, CimpleG.10, running in a single core took a little under 5 min to execute.

#### EpiDISH

EpiDISH (Epigenetic Dissection of Intra-Sample Heterogeneity, v2.14.1) [21] is a method also specifically targeting the problem of cell mixture deconvolution. Although it allows for the selection of differentially methylated probes, it is more tailored for the use of its pre-generated whole blood reference datasets for deconvolution. For benchmarking purposes, we use EpiDISH with default parameters and we use as reference their *centDHSbloodDMC.m* dataset. In this dataset, EpiDISH leverages the information of DNAse Hypersensitive Sites along with other purified blood cells data, from the Reinius et al. study [58], generated with the Illumina Infinium Human Methylation 450k platform to improve their deconvolution performance. EpiDISH uses by default the RPC algorithm for deconvolution, which is used in our evaluation. As previously mentioned, it supports three different algorithms RPC, Cibersort and CP.

#### minfi

The R package minfi (v1.44.0) is an all encompassing package for the analysis of DNAm data generated with the Illumina Infinium Human Methylation BeadChips [15]. For the deconvolution of cell mixtures, it provides the function *estimateCellCounts* [25]. It is important to note that, unlike other methods, minfi requires a specific type of object to perform their deconvolution estimations. This object is an *RGChannelSet*, a specific object from this package that is basically devoid of any pre-processing. By default, during computation, *estimateCellCounts* will normalize the data, applying quantile normalization. This step normalizes the user data along with minfi’s reference data. It uses quantile normalization, by default, for the type of data that we are analyzing, blood cells. However to make normalization more consistent with other data sets evaluated here, we opted to set the parameter controlling this, “processMethod,” to preprocessNoob. This approach was used in all of minfi’s related benchmarking analysis.

To perform its predictions, minfi uses by default a pre-generated reference dataset for deconvolution, “FlowSorted.Blood.450k.” This dataset is based on samples assayed as part of the Reinius et al. study [58] as well. It is provided in the form of an R package [25] and it needs to be installed prior to running minfi to deconvolve the data. To perform deconvolution it first computes which probes would be best to perform this task. By default, for the type of data in this study, it selects the 100 probes with the highest magnitude in effect between contrasts (50 hyper- and 50 hypo-methylated) as selected by its F-stat p-value threshold ($p < 1e^{-8}$). Then, to compute the actual inferences, it uses the selected probes combined with an implementation of the CP algorithm described in Houseman et al. [26].

#### ENmix

ENmix (v1.34.0) is, similarly to minfi, an all encompassing package for the analysis of DNAm data from the Illumina Infinium Human Methylation platform [18]. For the deconvolution of cell mixtures it provides the function *estimateCellProp*. It uses a pre-generated reference dataset “FlowSorted.Blood.450k” and uses the same DNAm selection procedure and deconvolutuion algorithm (CP) as minfi. It is more flexible than minfi, as it allows the use of pre-processed DNAm data (any matrix representation with methylation values) and it allows the user to choose if quantile normalization should be applied or not. We have set the parameter controlling normalization (normalize) to “FALSE”. More importantly, it allows the user to pick the number of probes with the highest magnitude in effect (hyper- and hypo-methylated) to be used for deconvolution. We should note that although ENmix allows you to set this parameter (nProbes) to 1, their implementation makes it so that it will always select 1 probe which is hyper-methylated and 1 probe which is hypo-methylated, effectively doubling the number of probes it is actually selecting. If nProbes is set to 50, the selection of probes should mirror the selection done by minfi.

### Benchmark data for the cell deconvolution problem

To evaluate the performance of the different methods for deconvolution we used data compiled in Salas et al. [27]. This includes a reference data set with 12 purified cell types, which is used for cell signature detection. For deconvolution, we explore here two artificial and one real leukocyte mixture datasets with fluorescence-activated cell sorting (FACS) data associated to it. One note, training IDOL requires an additional mixture data set (see IDOL description above), which is not used for benchmarking to avoid bias.

#### Purified 12 leukocytes data

This dataset is a subset of the data with the GEO accession number GSE167998. Comprising 56 different purified leukocyte samples spanning across 12 different leukocyte subtypes (Additional file 2: Table S4). These leukocyte subtypes are neutrophils (neu), eosinophils (eos), basophils (bas), monocytes (mono), B naive cells (bnv), B memory cells (bmem), CD4 T naive cells (cd4nv), CD4 T memory cells (cd4mem), T regulatory cells (treg), CD8 T naive cells (cd8nv), CD8 T memory cells (cd8mem), and natural killer cells (nk).

To process these data, we have acquired the raw data (.IDAT files) from GEO. We have pre-processed the data with the data processing pipeline from SeSAMe [19] (*openSesame* function, sesame R package v1.16.1), setting the pre-processing code argument to “QGCDPB.” With this setting, the pipeline will perform the following tasks in this order: mask probes of poor design, mask all but cg-probes, infer channel for Infinium-I probes, apply non-linear dye-bias correction, perform detection *p*-value masking using out-of-band probes and finally perform background subtraction using out-of-band probes (often called noob normalization). After pre-processing, we removed the masked probes from our data. Further, using Illumina’s EPIC platform annotation, we have removed probes associated with sexual chromosomes. In the end we have a matrix with beta-values for 56 samples across 689,105 probes.

#### Mixture Leukocyte dataset 1

This dataset with GEO accession number GSE182379, contains a total of 12 different artificially-mixed-leukocyte samples, with the same cell types as in the “Purified 12 leukocytes data” above (Additional file 2: Table S5). For this dataset we have downloaded the processed data from GEO. In the end we have a matrix with beta-values for 12 samples across 865,859 probes.

In the experiments where we needed to combine the leukocyte subsets into larger groups, we simply summed up the predictions (or the true values) of the more refined subsets into the combined groups. We have combined neutrophils, eosinophils and basophils into granulocytes (gran); B naive cells and B memory cells into B cells (bcells); CD4 T naive cells, CD4 T memory cells and T regulatory cells into CD4 T cells (cd4t); and finally, CD8 T naive cells and CD8 T memory cells into CD8 T cells. Monocytes and NK cells were unaffected by this change. For simplicity, we refer to this dataset as Leukocyte dataset 1.

#### Mixture Leukocyte dataset 2

This dataset is a subset of the data with the GEO accession number GSE167998. It includes 6 samples of artificially mixed leukocytes (Additional file 2: Table S6). Twelve different leukocyte subtypes were used to produce these mixes. The same pre-processing pipeline was used as for the “Purified 12 leukocytes data” (see above). In the end we have a matrix with beta-values for 6 samples across 689,105 probes. The same procedure to combine leukocyte subsets into larger groups applied in Leukocyte dataset 1 was also applied to this dataset. For simplicity, we refer to this dataset as Leukocyte dataset 2.

#### Mixture Leukocyte dataset 3

This dataset is a subset of the data with the GEO accession number GSE110530. In total this dataset contains 12 samples, however only 5 of these contain partial FACS information. These are the samples we use for our evaluation (Additional file 2: Table S7). These samples are real blood samples from a male adult volunteer taken across different points in time. The same pre-processsing pipeline was used as for the “Purified 12 leukocytes data” (see above). In the end we have a matrix with beta-values for 5 samples across 729,973 probes.

Notably and in contrast with the other datasets, the FACS information available for these samples only covers 5 different cell types. These are CD4 T cells (cd4t), CD8 T cells (cd8t), granulocytes (gran) and monocytes (mono). The last cell type was the CD3- fraction of lymphocytes and it is represented as being the sum of B and NK cells (nont_b_and_nk). Given this, for the predictions made for these samples, we have summed up the cd4t, cd8t and gran groups as described before (see Leukocyte dataset 2). Furthermore, we have summed up bcells and nk into the nont_b_and_nk group.

### Experimental design for cell deconvolution

The objective of this experiment is to evaluate different deconvolution methods under different datasets. Some of these methods need to be trained *a priori* to create a deconvolution reference matrix (*W* in Eq. 5), while others use pre-generated deconvolution reference datasets derived from other experimetns to perform their inferences.

First we focus on the methods that need to be trained. These are IDOL and CimpleG (and methods under their respective umbrellas). We train CimpleG, CimpleG.10, elasticnet (ElasticNet), boostedtree (XGBoost), neuralnet (Neural network), randforest (Random forests) and decisiontree (Decision tree) with the “Purified 12 leukocytes data” with default parameters. The exception being the train$\_$only parameter which was set to “TRUE” as this dataset is too small for a train-test split to be performed. Here, the cross-validation step described in the “CimpleG for signature selection” section is still performed. For CimpleG.10, the parameter “n_sigs” is set to 10 (instead of 1, the default). As mentioned previously, IDOL (and IDOLmin) need to be trained in two separate steps. The first training step, using the “Purified 12 leukocytes data” was performed with default parameters and is the same for both IDOL and IDOLmin. For the second step (optimization), the libSize parameter was set to 1200 for IDOL and 13 for IDOLmin, and the numCores parameters was set to 6 (default is 4) in order to speed up the computation time.

Next, we performed the cell deconvolution predictions by using default settings. We have capped predicted proportions to 0 in case negative values were present. We always generate predictions for the more granular classes (12 leukocyte subsets) for the methods that can do so (CimpleG and IDOL). We then sum up these inferences to accommodate the broader cell-type groups, 5 or 6 major leukocyte groups depending on the dataset.

We should note that, for most methods, the same signatures were used across deconvolution. The exceptions are minfi and ENmix, which perform feature selection at each deconvolution run and might select distinct signatures. Taking CimpleG as an example, for the deconvolution problem, CimpleG was trained once on the expanded leukocyte reference. Then this trained model was used to evaluate the three different leukocyte datasets as well as their more broadly annotated, simplified versions. ENmix and minfi however, due to the nature of using pre-generated reference datasets, and the implementation of their deconvolution algorithms, only really select which probes will be used for deconvolution at the time of deconvolution. This means for example, that ENmix.min can use two distinct sets of signatures when trying to deconvolve Leukocyte dataset 1 and Leukocyte dataset 2. Thus, with that caveat (marked with a $*$), we have in ascending order of number of signatures required for deconvolution the methods: CimpleG (12), IDOL.min (13), ENmix.min (24$*$), minfi and ENmix (100$*$), CimpleG.10 (120), EpiDISH (333), and IDOL (1200).

To quantitatively evaluate the performance of different methods for deconvolution we compute the root mean squared error (RMSE, Eq. 6) and two different versions of the R-squared metric. The R-squared ($R^2$, Eq. 7) [42, 43], and the R-squared of the fit ($R^2(fit)$, Eq. 10). We compute the RMSE as:

6 $$\begin{aligned} RMSE = \sqrt{\frac{\sum _{i=1}^{n}(true_i - pred_i)^2}{n}} \end{aligned}$$

where $true_i$ are the true values of sample *i*, $pred_i$ are the predicted values for sample *i* and *n* is the total number of samples.

We compute $R^2(diagonal)$ as:

7 $$\begin{aligned} R^2 = 1 - \frac{\sum _{i=1}^{n}(true_i - pred_i)^2}{\sum _{i=1}^{n}(true_i - \overline{true})^2} \end{aligned}$$

where $true_i$ are the true values of sample *i*, $\overline{true}$ is the mean of all true values, $pred_i$ are the predicted values for sample *i* and *n* is the total number of samples. Given the linear model fit $\textrm{T} = a * pred + b$ estimated by the deconvolution model, we first estimate the residuals

8 $$\begin{aligned} res_i = true_i - \textrm{T}(pred_i), \end{aligned}$$

and the $R^2$(fit) can be defined as:

9 $$\begin{aligned} R^2 = 1 - \frac{\sum _{i=1}^{n}(res_i)^2}{\sum _{i=1}^{n}(pred_i - \overline{pred_i})^2}. \end{aligned}$$

Finally, the statistic is adjusted to consider sample size:

10 $$\begin{aligned} R^2(fit) = R_{adj}^2 = 1 - (1 - R^2)\frac{n-1}{n-p-1}. \end{aligned}$$

where *n* is the number of samples and *p* is the number of variables used for the linear fit. The value of Eq. 10 approaches that of Eq. 9, the higher the number of samples is. This is, in short, the procedure done when trying to calculate the $R^2$ using the functions *lm* and *summary* in R (the programming language).

In this study we also refer to these metrics as $R^2$ diagonal and $R^2$ fitted for ease of understanding. We compute these $R^2$ metrics because their use is common place, however we argue that these should not be employed for the evaluation of deconvolution results (see Discussion). To rank the performance of each method is in comparison to its peers, we use the Friedman and Nemenyi post hoc test. This test ranks and evaluates, for each given metric, if the performance of a given method is significantly worse than the performance of the others.

## Supplementary information

**Additional file 1:** **Supplementary Figures S1** **to** **S11**.

**Additional file 2:** **Supplementary Tables S1** **to** **S7.** This work includes 7 supplementary tables. **Table S1** has information regarding CimpleG signatures for classification. **Table S2** has information of data sets used for the somatic cells data set. **Table S3** has information of data sets used for the leukocyte cells data set. **Table S4** has information of data sets used for the purified 12 Leukocyte Data. **Table S5** has information of data sets used for the mixture leukocyte dataset 1. **Table S6** has information of data sets used for the mixture leukocyte dataset 2. **Table S7** has information of data sets used for the mixture leukocyte dataset 3.

**Additional file 3.** Review history.

## Data Availability

The following datasets from the Gene Expression Omnibus (GEO, https://www.ncbi.nlm.nih.gov/geo/) have been used in this study: GSE103253 [61], GSE107226 [62], GSE40699 [63], GSE41933 [64], GSE43976 [65], GSE50222 [66], GSE52025 [67], GSE52112 [68], GSE58622 [69], GSE59065 [70], GSE59091 [71], GSE59250 [72], GSE59796 [73], GSE60753 [74], GSE63409 [75], GSE65078 [76], GSE68134 [77], GSE71955 [78], GSE74877 [79], GSE77135 [80], GSE79144 [81], GSE79695 [82], GSE82234 [83], GSE85647 [84], GSE87095 [85], GSE87177 [86], GSE88824 [87], GSE92843 [88], GSE95096 [89], GSE98203 [90], GSE99716 [91], GSE104287 [92], GSE106099 [93], GSE109042 [94], GSE111396 [95], GSE122126 [45], GSE34486 [96], GSE51921 [97], GSE53302 [98], GSE68851 [99], GSE71244 [100], GSE74486 [101], GSE85566 [102], GSE86258 [103], GSE86829 [12], GSE87797 [104], GSE35069 [58], GSE49618 [105], GSE66562 [106], GSE110554 [107], GSE68456 [108], GSE167998 [27], GSE182379 [27], GSE110530 [109]. Detailed tables with which samples were used from which study as well as their purpose are present in the supplementary tables in Additional file 2: Tables S2-S7. Finally, we also include the datasets that we have gathered and curated to benchmark cell-type classification as discrete files ready to be used for analysis. These can be obtained from zenodo at https://doi.org/10.5281/zenodo.8047172 [110]

## Footnotes

1 For IDOL the minimum possible signature size is K classes + 1

## Code availability

CimpleG’s source code is licensed under GPL3.0 and it can be found at github.com/CostaLab/CimpleG. The version of CimpleG used in this manuscript and the code to perform the analysis can be found in zenodo at 10.5281/zenodo.8045496 [59] and 10.5281/zenodo.8045462 [60], respectively. A website with tutorials, examples, and documentation can be found at costalab.github.io/CimpleG.

## References

[1] Smith ZD, Meissner A (2013). DNA methylation: roles in mammalian development. Nat Rev Genet..

[2] Horvath S (2013). DNA methylation age of human tissues and cell types. Genome Biol..

[3] Horvath S, Raj K (2018). DNA methylation-based biomarkers and the epigenetic clock theory of ageing. Nat Rev Genet..

[4] Weidner C, Lin Q, Koch C, Eisele L, Beier F, Ziegler P (2014). Aging of blood can be tracked by DNA methylation changes at just three CpG sites. Genome Biol..

[5] Lin Q, Weidner CICI, Costa IGIG, Marioni RERE, Ferreira MRP, Deary IJ (2016). DNA methylation levels at individual age - associated CpG sites can be indicative for life expectancy. Aging..

[6] Teschendorff AE, Gao Y, Jones A, Ruebner M, Beckmann MW, Wachter DL (2016). DNA methylation outliers in normal breast tissue identify field defects that are enriched in cancer. Nat Commun..

[7] Widschwendter M, Jones A, Evans I, Reisel D, Dillner J, Sundström K (2018). Epigenome-based cancer risk prediction: rationale, opportunities and challenges. Nat Rev Clin Oncol..

[8] de Almeida DC, Ferreira MRP, Franzen J, Weidner CI, Frobel J, Zenke M (2016). Epigenetic Classification of Human Mesenchymal Stromal Cells. Stem Cell Rep..

[9] Salhab A, Nordström K, Gasparoni G, Kattler K, Ebert P, Ramirez F (2018). A comprehensive analysis of 195 DNA methylomes reveals shared and cell-specific features of partially methylated domains. Genome Biol..

[10] Koestler DC, Jones MJ, Usset J, Christensen BC, Butler RA, Kobor MS (2016). Improving cell mixture deconvolution by identifying optimal DNA methylation libraries (IDOL). BMC Bioinformatics..

[11] Teschendorff AE, Relton CL (2018). Statistical and integrative system-level analysis of DNA methylation data. Nat Rev Genet..

[12] Pidsley R, Zotenko E, Peters TJ, Lawrence MG, Risbridger GP, Molloy P (2016). Critical evaluation of the Illumina MethylationEPIC BeadChip microarray for whole-genome DNA methylation profiling. Genome Biol..

[13] Schmidt M, Maié T, Dahl E, Costa IG, Wagner W (2020). Deconvolution of cellular subsets in human tissue based on targeted DNA methylation analysis at individual CpG sites. BMC Biol..

[14] Wagner W. How to Translate DNA Methylation Biomarkers Into Clinical Practice. Front Cell Dev Biol. 2022;10. 10.3389/FCELL.2022.854797. 10.3389/fcell.2022.854797 PMC8905294 35281115

[15] Aryee MJ, Jaffe AE, Corrada-Bravo H, Ladd-Acosta C, Feinberg AP, Hansen KD (2014). Minfi: A flexible and comprehensive Bioconductor package for the analysis of Infinium DNA methylation microarrays. Bioinformatics..

[16] Lafta MH, Gangadhar L. RNBeads 2.0: Comprehensive analysis of DNA methylation data. Ann Trop Med Public Health. 2019;19(Special Issue):2003–19.

[17] Tian Y, Morris TJ, Webster AP, Yang Z, Beck S, Feber A (2017). ChAMP: Updated methylation analysis pipeline for Illumina BeadChips. Bioinformatics..

[18] Xu Z, Niu L, Li L, Taylor JA (2016). ENmix: a novel background correction method for Illumina HumanMethylation450 BeadChip. Nucleic Acids Res..

[19] Zhou W, Triche TJ, Laird PW, Shen H (2018). SeSAMe: reducing artifactual detection of DNA methylation by Infinium BeadChips in genomic deletions. Nucleic Acids Res..

[20] Smyth GK (2004). Linear Models and Empirical Bayes Methods for Assessing Differential Expression in Microarray Experiments. Stat Appl Genet Mol Biol..

[21] Teschendorff AE, Breeze CE, Zheng SC, Beck S (2017). A comparison of reference-based algorithms for correcting cell-type heterogeneity in Epigenome-Wide Association Studies. BMC Bioinformatics..

[22] Zou H, Hastie T (2005). Regularization and variable selection via the elastic net. J R Stat Soc Ser B Stat Methodol..

[23] Friedman J, Hastie T, Tibshirani R (2010). Regularization Paths for Generalized Linear Models via Coordinate Descent. J Stat Softw..

[24] Jurmeister P, Bockmayr M, Seegerer P, Bockmayr T, Treue D, Montavon G, et al. Machine learning analysis of DNA methylation profiles distinguishes primary lung squamous cell carcinomas from head and neck metastases. Sci Transl Med. 2019;11(509). 10.1126/scitranslmed.aaw8513. 10.1126/scitranslmed.aaw8513 31511427

[25] Jaffe AE, Irizarry RA (2014). Accounting for cellular heterogeneity is critical in epigenome-wide association studies. Genome Biol..

[26] Houseman EA, Accomando WP, Koestler DC, Christensen BC, Marsit CJ, Nelson HH (2012). DNA methylation arrays as surrogate measures of cell mixture distribution. BMC Bioinformatics..

[27] Salas LA, Zhang Z, Koestler DC, Butler RA, Hansen HM, Molinaro AM (2022). Enhanced cell deconvolution of peripheral blood using DNA methylation for high-resolution immune profiling. Nat Commun..

[28] Frobel J, Božić T, Lenz M, Uciechowski P, Han Y, Herwartz R (2018). Leukocyte Counts Based on DNA Methylation at Individual Cytosines. Clin Chem..

[29] Chen XW, Wasikowski M. FAST: A roc-based feature selection metric for small samples and imbalanced data classification problems. In: Proceedings of the ACM SIGKDD International Conference on Knowledge Discovery and Data Mining. 2008. p. 124–132. 10.1145/1401890.1401910.

[30] Breiman L. Random forests. Mach Learn. 2001;45:5–32.

[31] Chen T, Guestrin C. XGBoost. Proceedings of the 22nd ACM SIGKDD International Conference on Knowledge Discovery and Data Mining. 2016. 10.1145/2939672.2939785.

[32] Pidsley R, Y Wong CC, Volta M, Lunnon K, Mill J, Schalkwyk LC. A data-driven approach to preprocessing Illumina 450K methylation array data. BMC Genomics. 2013;14(1). 10.1186/1471-2164-14-293. 10.1186/1471-2164-14-293 PMC3769145 23631413

[33] Whalen S, Schreiber J, Noble WS, Pollard KS. Navigating the pitfalls of applying machine learning in genomics. Nat Rev Genet. 2021;0123456789. 10.1038/s41576-021-00434-9. 10.1038/s41576-021-00434-9 34837041

[34] Kuhn M, Wickham H. Tidymodels: a collection of packages for modeling and machine learning using tidyverse principles. 2020. https://www.tidymodels.org. Accessed 13 June 2023.

[35] Gao J, Simon M (2005). Identification of a Novel Keratinocyte Retinyl Ester Hydrolase as a Transacylase and Lipase. J Investig Dermatol..

[36] Kang Y, Massagué J (2004). Epithelial-Mesenchymal Transitions: Twist in Development and Metastasis. Cell..

[37] Sjöstedt E, Zhong W, Fagerberg L, Karlsson M, Mitsios N, Adori C, et al. An atlas of the protein-coding genes in the human, pig, and mouse brain. Science. 2020;367. 10.1126/science.aay5947. 10.1126/science.aay5947 32139519

[38] Mullen KM, van Stokkum IHM. NNLS: The Lawson-Hanson algorithm for non-negative least squares (NNLS). 2012. R package version 1.4. https://CRAN.R-project.org/package=nnls. Accessed 13 June 2023.

[39] Cobos FA, Alquicira-Hernandez J, Powell JE, Mestdagh P, Preter KD (2020). Benchmarking of cell type deconvolution pipelines for transcriptomics data. Nat Commun..

[40] Sontag S, Bocova L, Hubens WHG, Nüchtern S, Schnitker M, Look T, et al. Toward Clinical Application of Leukocyte Counts Based on Targeted DNA Methylation Analysis. Clin Chem. 2022;68(5):646–56. 10.1093/clinchem/hvac006. https://academic.oup.com/clinchem/article-pdf/68/5/646/43752834/hvac006.pdf. 10.1093/clinchem/hvac006 35157041

[41] Baron U, Werner J, Schildknecht K, Schulze JJ, Mulu A, Liebert UG, et al. Epigenetic immune cell counting in human blood samples for immunodiagnostics. Sci Transl Med. 2018;10(452):eaan3508. 10.1126/scitranslmed.aan3508. https://www.science.org/doi/abs/10.1126/scitranslmed.aan3508. Accessed 13 June 2023. 10.1126/scitranslmed.aan3508 30068569

[42] Alexander DLJ, Tropsha A, Winkler DA (2015). Beware of R2: Simple, Unambiguous Assessment of the Prediction Accuracy of QSAR and QSPR Models. J Chem Inf Model..

[43] Kvalseth TO (1985). Cautionary Note about R 2. Am Stat..

[44] Lena PD, Sala C, Prodi A, Nardini C (2020). Methylation data imputation performances under different representations and missingness patterns. BMC Bioinformatics..

[45] Moss J, Magenheim J, Neiman D, Zemmour H, Loyfer N, Korach A (2018). Comprehensive human cell-type methylation atlas reveals origins of circulating cell-free DNA in health and disease. Nat Commun..

[46] Jupp S, Burdett T, Leroy C, Parkinson HE. A new Ontology Lookup Service at EMBL-EBI. SWAT4LS. 2015;2:118–119.

[47] Du P, Zhang X, Huang CC, Jafari N, Kibbe WA, Hou L (2010). Comparison of Beta-value and M-value methods for quantifying methylation levels by microarray analysis. BMC Bioinformatics..

[48] Bibikova M, Lin Z, Zhou L, Chudin E, Garcia EW, Wu B (2006). High-throughput DNA methylation profiling using universal bead arrays. Genome Res..

[49] Davis J, Goadrich M. The relationship between Precision-Recall and ROC curves. In: Proceedings of the 23rd international conference on Machine learning - ICML ’06. New York, New York, USA: ACM Press; 2006. p. 233–240. 10.1145/1143844.1143874. http://portal.acm.org/citation.cfm?doid=1143844.1143874. Accessed 13 June 2023.

[50] Edgar R, Domrachev M, Lash AE (2002). Gene Expression Omnibus: NCBI gene expression and hybridization array data repository. Nucleic Acids Res..

[51] Barrett T, Wilhite SE, Ledoux P, Evangelista C, Kim IF, Tomashevsky M, et al. NCBI GEO: archive for functional genomics data sets—update. Nucleic Acids Res. 2013;1(41):D991–5. 10.1093/NAR/GKS1193. 10.1093/nar/gks1193 PMC3531084 23193258

[52] Therneau T, Atkinson B. rpart: Recursive Partitioning and Regression Trees. 2022. R package version 4.1.16. https://CRAN.R-project.org/package=rpart. Accessed 13 June 2023.

[53] Wright MN, Ziegler A. ranger: A Fast Implementation of Random Forests for High Dimensional Data in C++ and R. J Stat Softw. 2017;77(1):1–17. 10.18637/jss.v077.i01.

[54] Chen T, He T, Benesty M, Khotilovich V, Tang Y, Cho H, et al.. xgboost: Extreme Gradient Boosting. 2021. R package version 1.5.0.2. https://CRAN.R-project.org/package=xgboost. Accessed 13 June 2023.

[55] Venables WN, Ripley BD. Modern Applied Statistics with S. 4th ed. New York: Springer; 2002.

[56] Sra S, Dhillon I. Generalized nonnegative matrix approximations with Bregman divergences. Adv Neural Inf Process Syst. 2005;18.

[57] Newman AM, Liu CL, Green MR, Gentles AJ, Feng W, Xu Y (2015). Robust enumeration of cell subsets from tissue expression profiles. Nat Methods..

[58] Reinius LE, Acevedo N, Joerink M, Pershagen G, Dahlén SE, Greco D (2012). Differential DNA methylation in purified human blood cells: implications for cell lineage and studies on disease susceptibility. PLoS ONE..

[59] Maié T. CimpleG, an R package to find (simple) CpG signatures. Github. 10.5281/zenodo.8045495.

[60] Maié T. CimpleG manuscript analysis. Zenodo. 10.5281/zenodo.8045462.

[61] Herzog EM. Early-and late-onset preeclampsia and the tissue-specific epigenome of the placenta and newborn. Gene Expression Omnibus. https://www.ncbi.nlm.nih.gov/geo/query/acc.cgi?acc=GSE103253. Accessed 13 June 2023. 10.1016/j.placenta.2017.08.070 28962690

[62] Lee JU. Global DNA methylation pattern of fibroblasts in idiopathic pulmonary fibrosis. Gene Expression Omnibus. https://www.ncbi.nlm.nih.gov/geo/query/acc.cgi?acc=GSE107226. Accessed 13 June 2023.

[63] Consortium EP, et al.. An integrated encyclopedia of DNA elements in the human genome. Gene Expression Omnibus. https://www.ncbi.nlm.nih.gov/geo/query/acc.cgi?acc=GSE40699. Accessed 13 June 2023.

[64] Reinisch A. Epigenetic and in vivo comparison of diverse MSC sources reveals an endochondral signature for human hematopoietic niche formation. Gene Expression Omnibus. https://www.ncbi.nlm.nih.gov/geo/query/acc.cgi?acc=GSE41933. Accessed 13 June 2023. 10.1182/blood-2014-04-572255 PMC4287636 25406351

[65] Marabita F. An evaluation of analysis pipelines for DNA methylation profiling using the Illumina HumanMethylation450 BeadChip platform. Gene Expression Omnibus. https://www.ncbi.nlm.nih.gov/geo/query/acc.cgi?acc=GSE43976. Accessed 13 June 2023. 10.4161/epi.24008 PMC3669124 23422812

[66] Nestor CE. DNA methylation changes separate allergic patients from healthy controls and may reflect altered CD4+ T-cell population structure. Gene Expression Omnibus. https://www.ncbi.nlm.nih.gov/geo/query/acc.cgi?acc=GSE50222. Accessed 13 June 2023. 10.1371/journal.pgen.1004059 PMC3879208 24391521

[67] Wagner JR. The relationship between DNA methylation, genetic and expression inter-individual variation in untransformed human fibroblasts. Gene Expression Omnibus. https://www.ncbi.nlm.nih.gov/geo/query/acc.cgi?acc=GSE52025. Accessed 13 June 2023. 10.1186/gb-2014-15-2-r37 PMC4053980 24555846

[68] Fernández AF. H3K4me1 marks DNA regions hypomethylated during aging in human stem and differentiated cells. Gene Expression Omnibus. https://www.ncbi.nlm.nih.gov/geo/query/acc.cgi?acc=GSE52112. Accessed 13 June 2023.

[69] Dahlman I. The fat cell epigenetic signature in post-obese women is characterized by global hypomethylation and differential DNA methylation of adipogenesis genes. Gene Expression Omnibus. https://www.ncbi.nlm.nih.gov/geo/query/acc.cgi?acc=GSE58622. Accessed 13 June 2023. 10.1038/ijo.2015.31 25783037

[70] Tserel L. Age-related profiling of DNA methylation in CD8+ T cells reveals changes in immune response and transcriptional regulator genes. Gene Expression Omnibus. https://www.ncbi.nlm.nih.gov/geo/query/acc.cgi?acc=GSE59065. Accessed 13 June 2023. 10.1038/srep13107 PMC4541364 26286994

[71] Butcher LM. Non-CG DNA methylation is a biomarker for assessing endodermal differentiation capacity in pluripotent stem cells. Gene Expression Omnibus. https://www.ncbi.nlm.nih.gov/geo/query/acc.cgi?acc=GSE59091. Accessed 13 June 2023. 10.1038/ncomms10458 PMC4740175 26822956

[72] Absher DM. Genome-wide DNA methylation analysis of systemic lupus erythematosus reveals persistent hypomethylation of interferon genes and compositional changes to CD4+ T-cell populations. Gene Expression Omnibus. https://www.ncbi.nlm.nih.gov/geo/query/acc.cgi?acc=GSE59250. Accessed 13 June 2023. 10.1371/journal.pgen.1003678 PMC3738443 23950730

[73] Zhang X. DNA methylation dynamics during ex vivo differentiation and maturation of human dendritic cells. Gene Expression Omnibus. https://www.ncbi.nlm.nih.gov/geo/query/acc.cgi?acc=GSE59796. Accessed 13 June 2023. 10.1186/1756-8935-7-21 PMC4144987 25161698

[74] Hlady RA. Epigenetic signatures of alcohol abuse and hepatitis infection during human hepatocarcinogenesis. Gene Expression Omnibus. https://www.ncbi.nlm.nih.gov/geo/query/acc.cgi?acc=GSE60753. Accessed 13 June 2023. 10.18632/oncotarget.2444 PMC4253444 25294808

[75] Jung N. An LSC epigenetic signature is largely mutation independent and implicates the HOXA cluster in AML pathogenesis. Gene Expression Omnibus. https://www.ncbi.nlm.nih.gov/geo/query/acc.cgi?acc=GSE63409. Accessed 13 June 2023. 10.1038/ncomms9489 PMC4633733 26444494

[76] Burrows CK. Genetic variation, not cell type of origin, underlies the majority of identifiable regulatory differences in iPSCs. Gene Expression Omnibus. https://www.ncbi.nlm.nih.gov/geo/query/acc.cgi?acc=GSE65078. Accessed 13 June 2023. 10.1371/journal.pgen.1005793 PMC4727884 26812582

[77] Wang XM. Induced pluripotent stem cell models of Zellweger spectrum disorder show impaired peroxisome assembly and cell type-specific lipid abnormalities. Gene Expression Omnibus. https://www.ncbi.nlm.nih.gov/geo/query/acc.cgi?acc=GSE68134. Accessed 13 June 2023. 10.1186/s13287-015-0149-3 PMC4553005 26319495

[78] Limbach M. Epigenetic profiling in CD4+ and CD8+ T cells from Graves’ disease patients reveals changes in genes associated with T cell receptor signaling. Gene Expression Omnibus. https://www.ncbi.nlm.nih.gov/geo/query/acc.cgi?acc=GSE71955. Accessed 13 June 2023. 10.1016/j.jaut.2015.09.006 26459776

[79] Holm K. An integrated genomics analysis of epigenetic subtypes in human breast tumors links DNA methylation patterns to chromatin states in normal mammary cells. Gene Expression Omnibus. https://www.ncbi.nlm.nih.gov/geo/query/acc.cgi?acc=GSE74877. Accessed 13 June 2023. 10.1186/s13058-016-0685-5 PMC4770527 26923702

[80] Ivanov NA. Strong components of epigenetic memory in cultured human fibroblasts related to site of origin and donor age. Gene Expression Omnibus. https://www.ncbi.nlm.nih.gov/geo/query/acc.cgi?acc=GSE77135. Accessed 13 June 2023. 10.1371/journal.pgen.1005819 PMC4767228 26913521

[81] Do C. Mechanisms and disease associations of haplotype-dependent allele-specific DNA methylation. Gene Expression Omnibus. https://www.ncbi.nlm.nih.gov/geo/query/acc.cgi?acc=GSE79144. Accessed 13 June 2023. 10.1016/j.ajhg.2016.03.027 PMC4863666 27153397

[82] Von der Heide E. Molecular alterations in bone marrow mesenchymal stromal cells derived from acute myeloid leukemia patients. Gene Expression Omnibus. https://www.ncbi.nlm.nih.gov/geo/query/acc.cgi?acc=GSE79695. Accessed 13 June 2023. 10.1038/leu.2016.324 27833093

[83] Franzen J. Senescence-associated DNA methylation is stochastically acquired in subpopulations of mesenchymal stem cells. Gene Expression Omnibus. https://www.ncbi.nlm.nih.gov/geo/query/acc.cgi?acc=GSE82234. Accessed 13 June 2023. 10.1111/acel.12544 PMC5242294 27785870

[84] Mamrut S. Methylome and transcriptome profiling in Myasthenia Gravis monozygotic twins. Gene Expression Omnibus. https://www.ncbi.nlm.nih.gov/geo/query/acc.cgi?acc=GSE85647. Accessed 13 June 2023. 10.1016/j.jaut.2017.05.005 28549776

[85] Julià A. Epigenome-wide association study of rheumatoid arthritis identifies differentially methylated loci in B cells. Gene Expression Omnibus. https://www.ncbi.nlm.nih.gov/geo/query/acc.cgi?acc=GSE87095. Accessed 13 June 2023. 10.1093/hmg/ddx177 28475762

[86] Uehiro N. Circulating cell-free DNA-based epigenetic assay can detect early breast cancer. Gene Expression Omnibus. https://www.ncbi.nlm.nih.gov/geo/query/acc.cgi?acc=GSE87177. Accessed 13 June 2023. 10.1186/s13058-016-0788-z PMC5168705 27993161

[87] Kennedy DW. Critical evaluation of linear regression models for cell-subtype specific methylation signal from mixed blood cell DNA. Gene Expression Omnibus. https://www.ncbi.nlm.nih.gov/geo/query/acc.cgi?acc=GSE88824. Accessed 13 June 2023. 10.1371/journal.pone.0208915 PMC6301777 30571772

[88] Kiehl S. Epigenetic silencing of downstream genes mediated by tandem orientation in lung cancer. Gene Expression Omnibus. https://www.ncbi.nlm.nih.gov/geo/query/acc.cgi?acc=GSE92843. Accessed 13 June 2023. 10.1038/s41598-017-04248-w PMC5478622 28634396

[89] Oleksiewicz U. TRIM28 and interacting KRAB-ZNFs control self-renewal of human pluripotent stem cells through epigenetic repression of pro-differentiation genes. Gene Expression Omnibus. https://www.ncbi.nlm.nih.gov/geo/query/acc.cgi?acc=GSE95096. Accessed 13 June 2023. 10.1016/j.stemcr.2017.10.031 PMC5785758 29198826

[90] Kozlenkov A. DNA methylation profiling of human prefrontal cortex neurons in heroin users shows significant difference between genomic contexts of hyper-and hypomethylation and a younger epigenetic age. Gene Expression Omnibus. https://www.ncbi.nlm.nih.gov/geo/query/acc.cgi?acc=GSE98203. Accessed 13 June 2023. 10.3390/genes8060152 PMC5485516 28556790

[91] Takasawa K. DNA hypermethylation enhanced telomerase reverse transcriptase expression in human-induced pluripotent stem cells. Gene Expression Omnibus. https://www.ncbi.nlm.nih.gov/geo/query/acc.cgi?acc=GSE99716. Accessed 13 June 2023. 10.1007/s13577-017-0190-x 29103143

[92] Verma D. Anti-mycobacterial activity correlates with altered DNA methylation pattern in immune cells from BCG-vaccinated subjects. Gene Expression Omnibus. https://www.ncbi.nlm.nih.gov/geo/query/acc.cgi?acc=GSE104287. Accessed 13 June 2023. 10.1038/s41598-017-12110-2 PMC5615063 28951586

[93] Cvitic S. Human fetoplacental arterial and venous endothelial cells are differentially programmed by gestational diabetes mellitus, resulting in cell-specific barrier function changes. Gene Expression Omnibus. https://www.ncbi.nlm.nih.gov/geo/query/acc.cgi?acc=GSE106099. Accessed 13 June 2023. 10.1007/s00125-018-4699-7 PMC6182654 30091044

[94] Lussier AA. DNA methylation as a predictor of fetal alcohol spectrum disorder. Gene Expression Omnibus. https://www.ncbi.nlm.nih.gov/geo/query/acc.cgi?acc=GSE109042. Accessed 13 June 2023.

[95] Clifford RL. Altered DNA methylation is associated with aberrant gene expression in parenchymal but not airway fibroblasts isolated from individuals with COPD. Gene Expression Omnibus. https://www.ncbi.nlm.nih.gov/geo/query/acc.cgi?acc=GSE111396. Accessed 13 June 2023. 10.1186/s13148-018-0464-5 PMC5838860 29527240

[96] Brönneke S. DNA methylation regulates lineage-specifying genes in primary lymphatic and blood endothelial cells. Gene Expression Omnibus. https://www.ncbi.nlm.nih.gov/geo/query/acc.cgi?acc=GSE34486. Accessed 13 June 2023. 10.1007/s10456-012-9264-2 22434260

[97] Fernández-Santiago R. Aberrant epigenome in iPSC-derived dopaminergic neurons from Parkinson’s disease patients. Gene Expression Omnibus. https://www.ncbi.nlm.nih.gov/geo/query/acc.cgi?acc=GSE51921. Accessed 13 June 2023. 10.15252/emmm.201505439 PMC4693505 26516212

[98] Bigot A. Age-associated methylation suppresses SPRY1, leading to a failure of re-quiescence and loss of the reserve stem cell pool in elderly muscle. Gene Expression Omnibus. https://www.ncbi.nlm.nih.gov/geo/query/acc.cgi?acc=GSE53302. Accessed 13 June 2023. 10.1016/j.celrep.2015.09.067 26526994

[99] Vizoso M. Aberrant DNA methylation in non-small cell lung cancer-associated fibroblasts. Gene Expression Omnibus. https://www.ncbi.nlm.nih.gov/geo/query/acc.cgi?acc=GSE68851. Accessed 13 June 2023.

[100] Mamrut S. Integrative analysis of methylome and transcriptome in human blood identifies extensive sex-and immune cell-specific differentially methylated regions. Gene Expression Omnibus. https://www.ncbi.nlm.nih.gov/geo/query/acc.cgi?acc=GSE71244. Accessed 13 June 2023. 10.1080/15592294.2015.1084462 PMC4844205 26291385

[101] Mendioroz M. Trans effects of chromosome aneuploidies on DNA methylation patterns in human Down syndrome and mouse models. Gene Expression Omnibus. https://www.ncbi.nlm.nih.gov/geo/query/acc.cgi?acc=GSE74486. Accessed 13 June 2023.

[102] Nicodemus-Johnson J. DNA methylation in lung cells is associated with asthma endotypes and genetic risk. Gene Expression Omnibus. https://www.ncbi.nlm.nih.gov/geo/query/acc.cgi?acc=GSE85566. Accessed 13 June 2023. 10.1172/jci.insight.90151 PMC5139904 27942592

[103] Pidsley R. Enduring epigenetic landmarks define the cancer microenvironment. Gene Expression Omnibus. https://www.ncbi.nlm.nih.gov/geo/query/acc.cgi?acc=GSE86258. Accessed 13 June 2023. 10.1101/gr.229070.117 PMC5932604 29650553

[104] Fernandez-Rebollo E. Human platelet lysate versus fetal calf serum: these supplements do not select for different mesenchymal stromal cells. Gene Expression Omnibus. https://www.ncbi.nlm.nih.gov/geo/query/acc.cgi?acc=GSE87797. Accessed 13 June 2023. 10.1038/s41598-017-05207-1 PMC5506010 28698620

[105] Network CGAR. Genomic and epigenomic landscapes of adult de novo acute myeloid leukemia. Gene Expression Omnibus. https://www.ncbi.nlm.nih.gov/geo/query/acc.cgi?acc=GSE49618. Accessed 13 June 2023.

[106] Schlums H. Cytomegalovirus infection drives adaptive epigenetic diversification of NK cells with altered signaling and effector function. Gene Expression Omnibus. https://www.ncbi.nlm.nih.gov/geo/query/acc.cgi?acc=GSE66562. Accessed 13 June 2023. 10.1016/j.immuni.2015.02.008 PMC4612277 25786176

[107] Salas LA. FlowSorted.Blood.EPIC: An optimized library for reference-based deconvolution of whole-blood biospecimens assayed using the Illumina HumanMethylationEPIC BeadArray (II). Gene Expression Omnibus. https://www.ncbi.nlm.nih.gov/geo/query/acc.cgi?acc=GSE110554. Accessed 13 June 2023. 10.1186/s13059-018-1448-7 PMC5975716 29843789

[108] de Goede OM. Nucleated red blood cells impact DNA methylation and expression analyses of cord blood hematopoietic cells. Gene Expression Omnibus. https://www.ncbi.nlm.nih.gov/geo/query/acc.cgi?acc=GSE68456. Accessed 13 June 2023. 10.1186/s13148-015-0129-6 PMC4567832 26366232

[109] Salas LA. Longitudinal dataset: An optimized library for reference-based deconvolution of whole-blood biospecimens assayed using the Illumina HumanMethylationEPIC BeadArray (I). Gene Expression Omnibus. https://www.ncbi.nlm.nih.gov/geo/query/acc.cgi?acc=GSE110530. Accessed 13 June 2023. 10.1186/s13059-018-1448-7 PMC5975716 29843789

[110] Maié T. CimpleG benchmarking datasets. Zenodo. 10.5281/zenodo.8047172.

