# Supplementary material for: CimpleG: finding simple CpG methylation signatures
Source: Genome Biol. 2023 Jul 10;24:161. doi: 10.1186/s13059-023-03000-0 (PMC10332104; doi:10.1186/s13059-023-03000-0)
Supplement: Supplementary file 1 — Additional file 1: Supplementary Figures S1 to S11. [file 13059_2023_3000_MOESM1_ESM.pdf]

CimpleG: Finding simple CpG methylation signatures

Tiago Maié[1+], Marco Schmidt[2,3+], Myriam Erz[1], Wolfgang Wagner[2,3], and Ivan G. Costa[1*]

[1]Institute for Computational Genomics, Joint Research Center for Computational Biomedicine, RWTH Aachen University Medical School, 52074 Aachen, Germany

[2]Helmholtz Institute for Biomedical Engineering, RWTH Aachen University, 52074 Aachen, Germany

[3]Institute for Stem Cell Biology, RWTH Aachen University Medical School, 52074 Aachen, Germany

[*]corresponding authors: ivan.costa@rwth-aachen.de

[+]these authors contributed equally to this work

Supplementary material

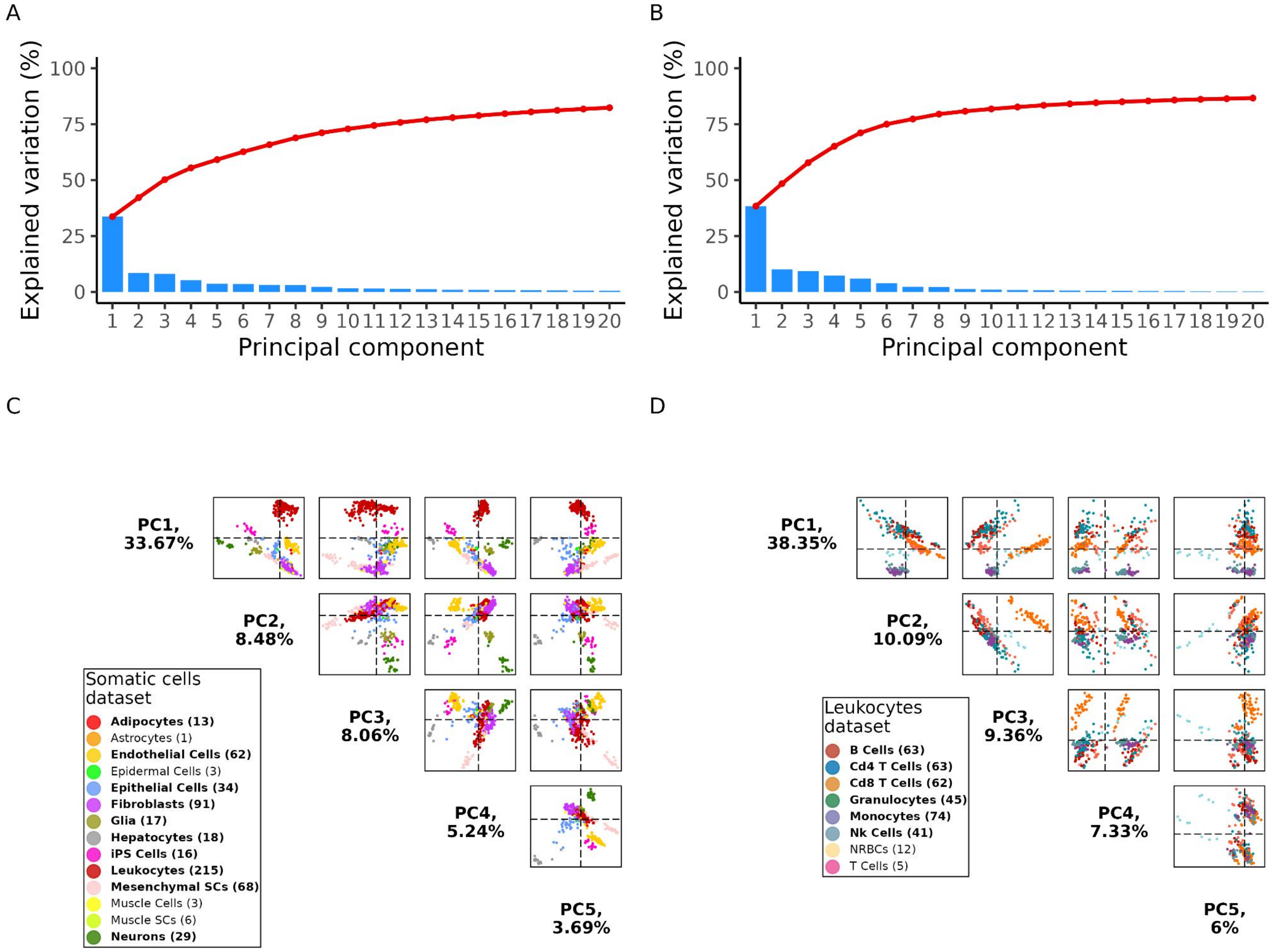

Fig. S1. Principal component analysis of the datasets used. (A-B) Scree plots from the PCA analysis for the somatic cells dataset and the leukocytes dataset respectively; (C-D) PCA pair-plots with the first 5 Principal Components for the somatic cells dataset and the leukocytes dataset respectively. The separation of individual cell types varies across the different principal components.

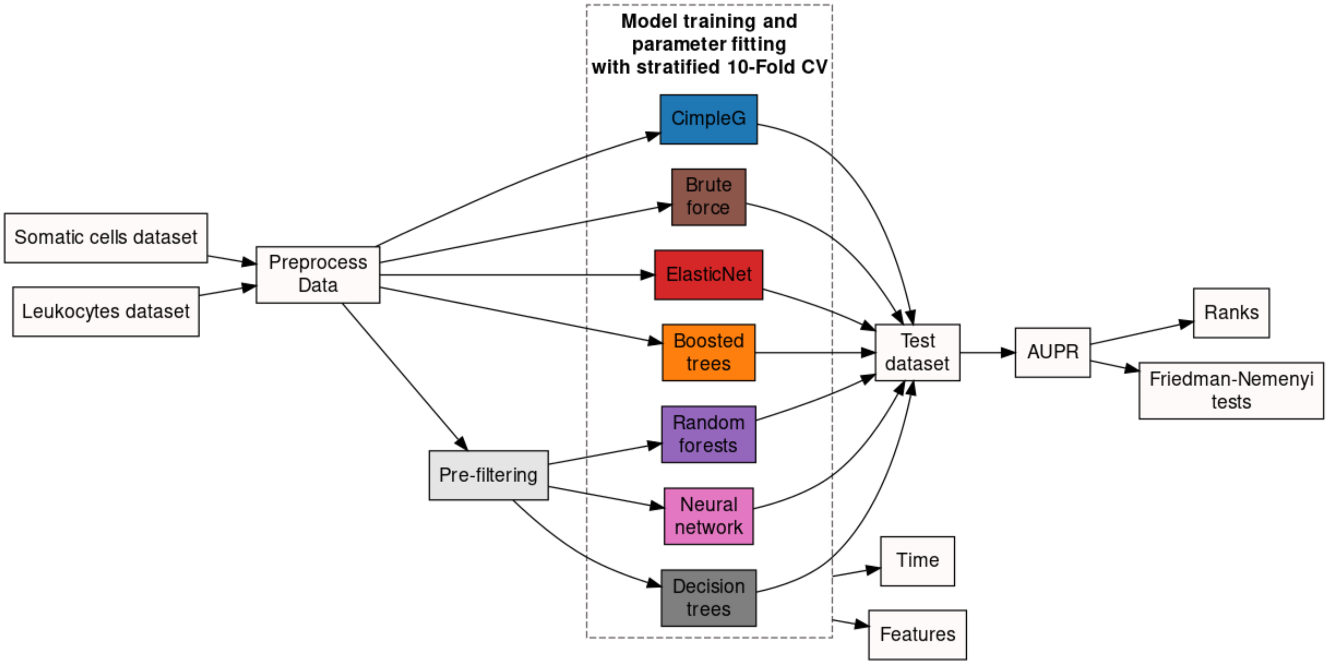

Fig. S2. Schematic representation of the experimental design used for benchmarking the cell-type classification problem.

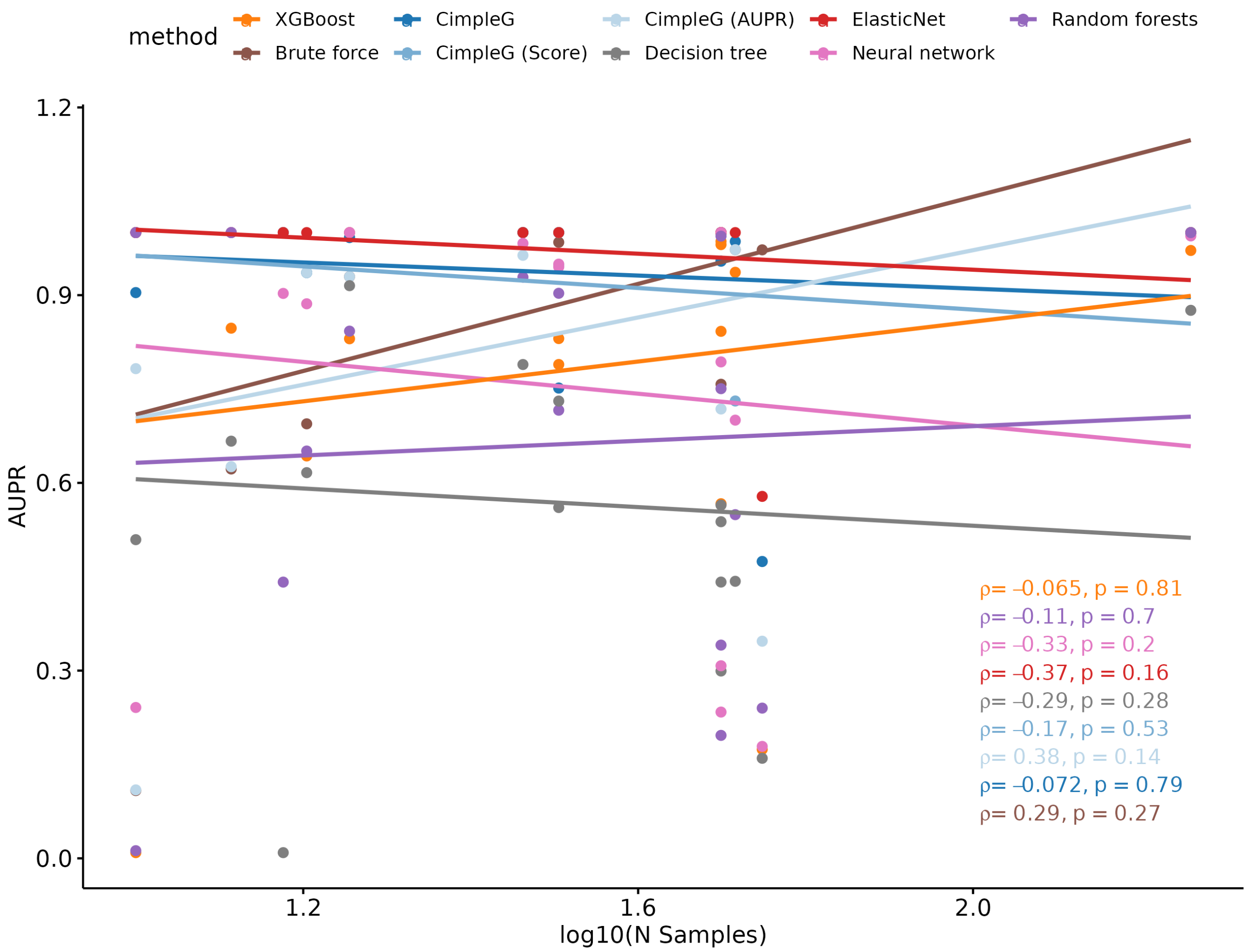

Fig. S3. Relationship between the Area Under the Precision–Recall curve (AUPR) and the number of samples for the target class (in the log10 scale) for all evaluated methods and datasets. Lines represent linear fits, which evaluate if the AUPR of distinct methods is related to the sample size of the target class. Statistics shown are Spearman's correlation coefficient rho and the associated p-value.

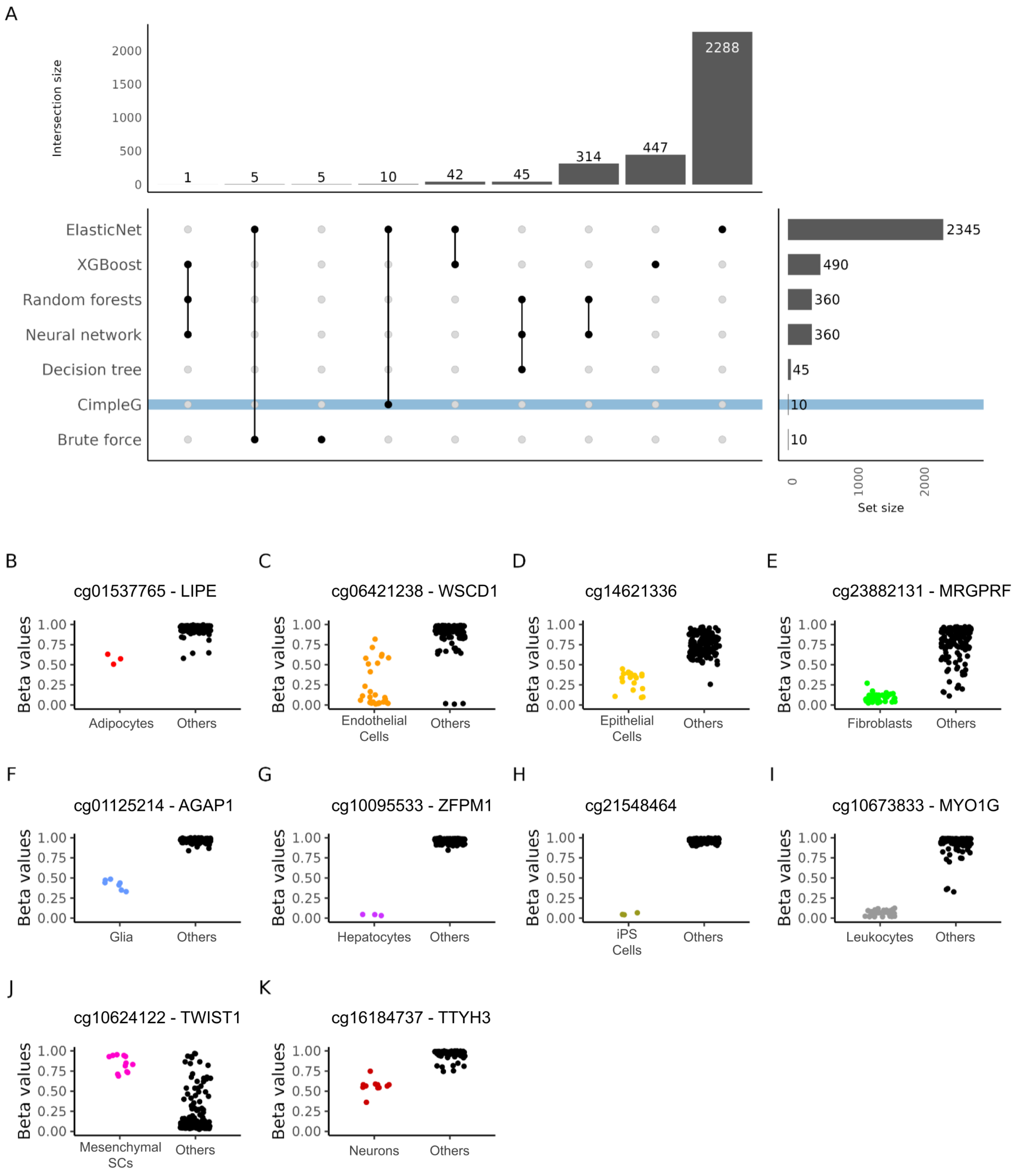

Fig. S4. Somatic cell signatures. (A) Upset plot showing the total number of selected DNAm sites per method (y-axis) and how these DNAm sites intersect for distinct combinations of method (x-axis) for the somatic cells dataset. Connected dots in a column indicate the combination of methods. (B-K) Beta values (y-axis) of CpG sites selected by CimpleG on the test data . The colour of the points corresponds to the target cell type, while points in black correspond to the cell types that are not the targets for that signature.

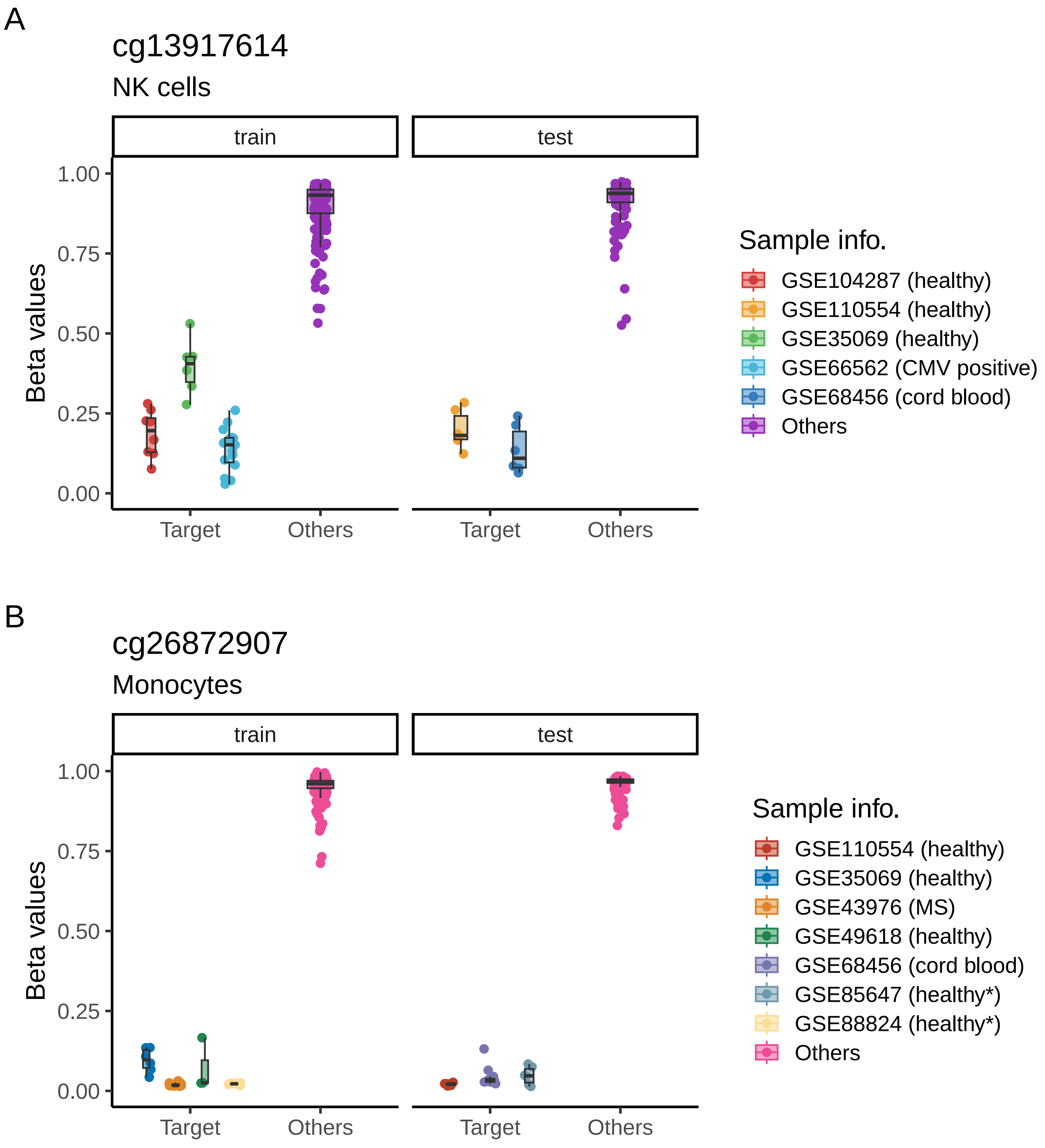

Fig. S5. Distribution of samples across train and test data for the leukocyte classification dataset for the (A) NK and (B) Monocytes signature. Samples across our datasets were selected in order to maximize sample size and allowing for some biological variability. To investigate if these affect DNAm values of CimpleG selected sites, we stratify samples according to batch, disease status (MS-multiple scelorsis and CVM - citomegalovirus) and origin (adult vs. cord blood). We observe that biological covariates, such as disease status and origin, did not influence the DNAm values of the CimpleG selected sites.

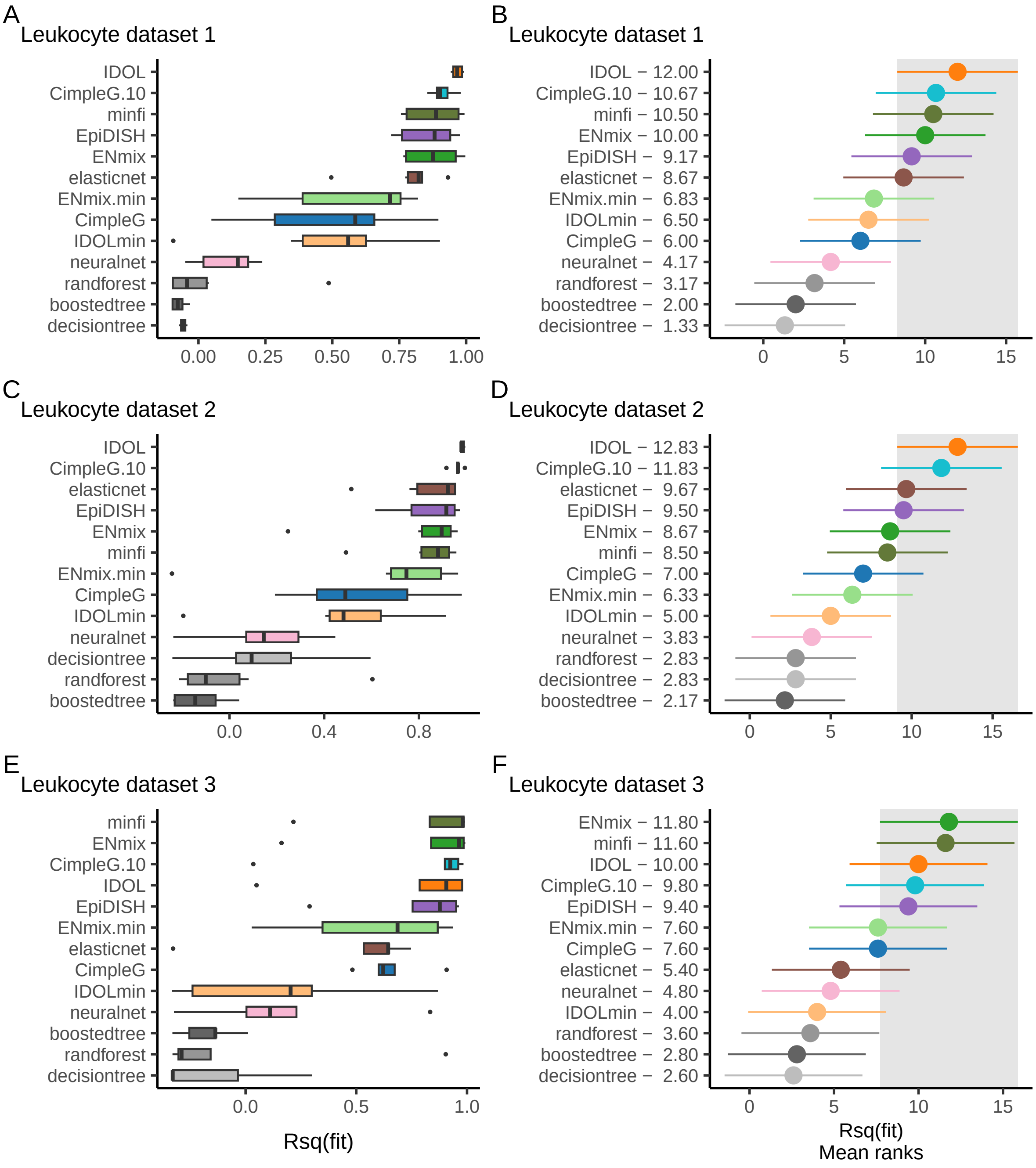

Fig. S6. R^2(fit) (left) and its mean ranks (right) are shown for each classifier/deconvolution method and for the Leukocyte 1 (A-B), 2 (C-D) and 3 (E-F) data sets with major leukocyte cell types. Methods are ranking from top-down regarding the best performance (highest R^2). For the R^2 mean rank plots, the best method, and its 95% confidence interval (Friedman and Nemenyi post-hoc test) is highlighted in grey. Methods whose average R^2 does not overlap at all with the highlighted area, are significantly worse than the top performing method.

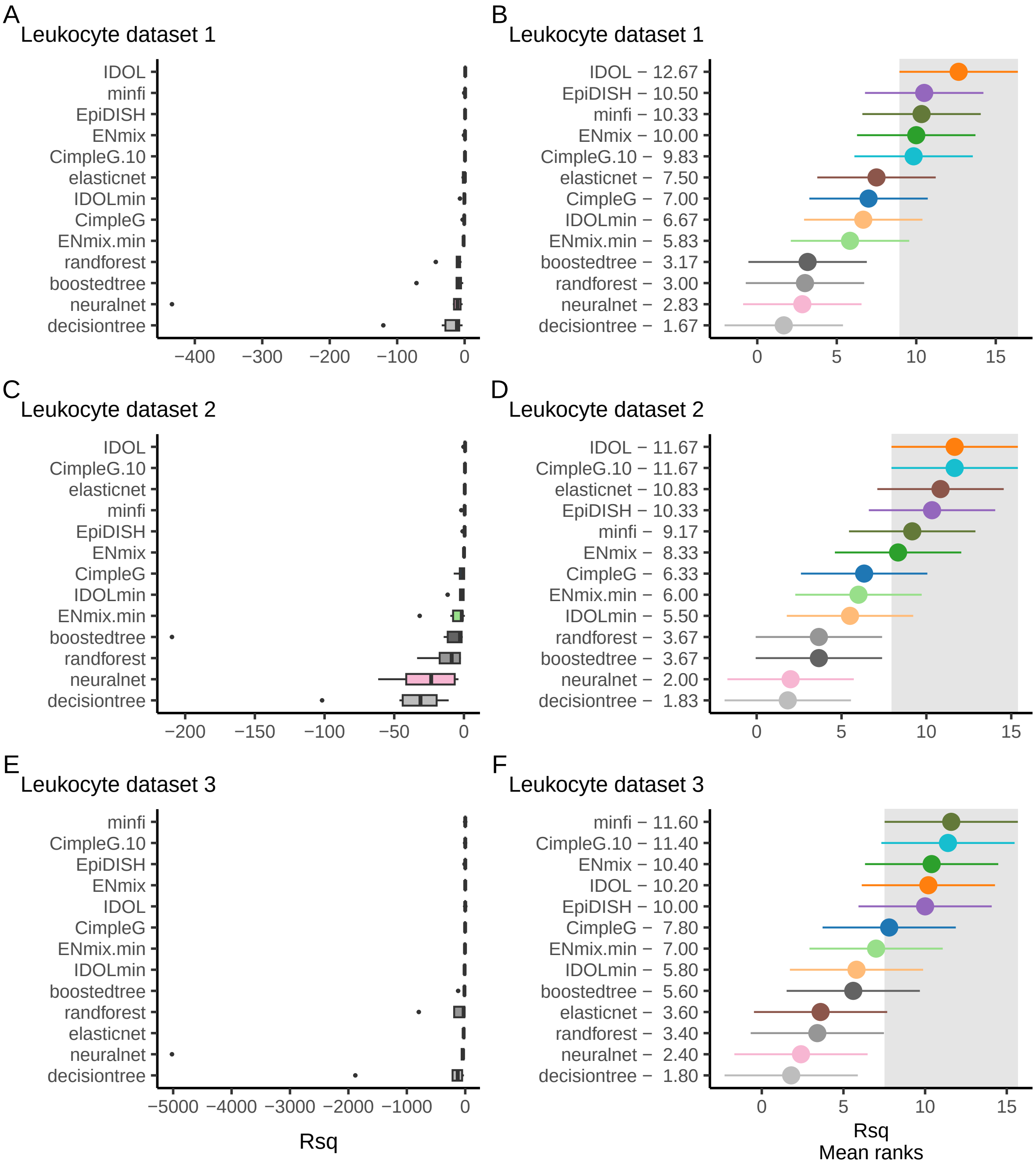

Fig. S7. R^2(diagonal) (left) and its mean ranks (right) are shown for each classifier/deconvolution method and for the Leukocyte 1 (A-B), 2 (C-D) and 3 (E-F) data sets with major leukocyte cell types. Methods are ranking from top-down regarding the best performance (highest R^2). For the R^2 mean rank plots, the best method, and its 95% confidence interval (Friedman and Nemenyi post-hoc test) is highlighted in grey. Methods whose average R^2 does not overlap at all with the highlighted area, are significantly worse than the top performing method.

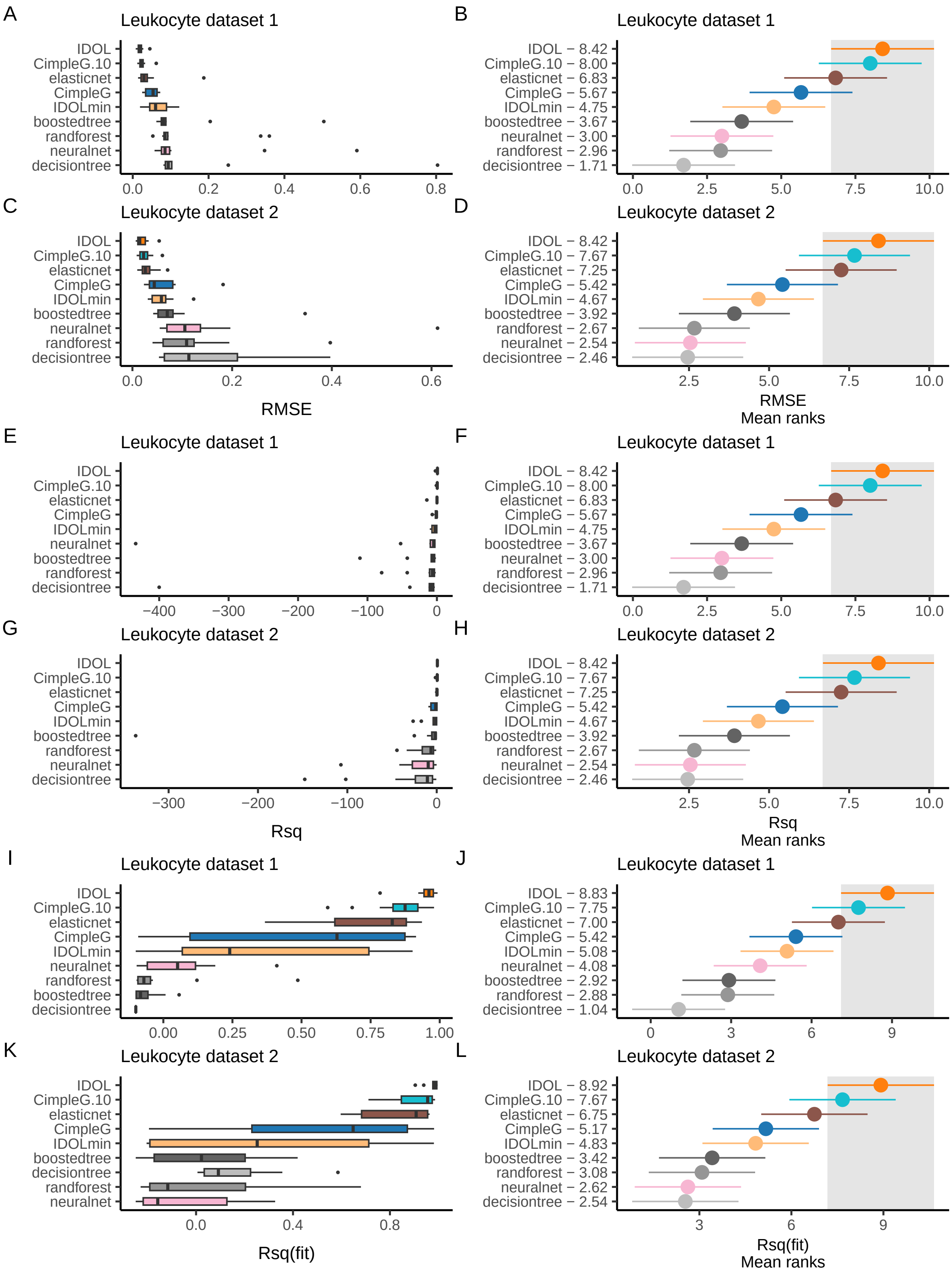

Fig. S8. RMSE (left) and its mean ranks (right) are shown for each classifier/deconvolution method and for the Leukocyte 1 (A-B) and 2 (C-D) with 12 leukocyte cell types. Methods are ranking from top-down regarding the best performance (highest RMSE). For the RMSE mean rank plots, the best method, and its 95% confidence interval (Friedman and Nemenyi post-hoc test) is highlighted in grey. Methods whose average RMSE does not overlap at all with the highlighted area, are significantly worse than the top performing method. Panels (E-H) and (I-L) are similar to (A-D) but based on R2 diagonal and R2 fit statistics.

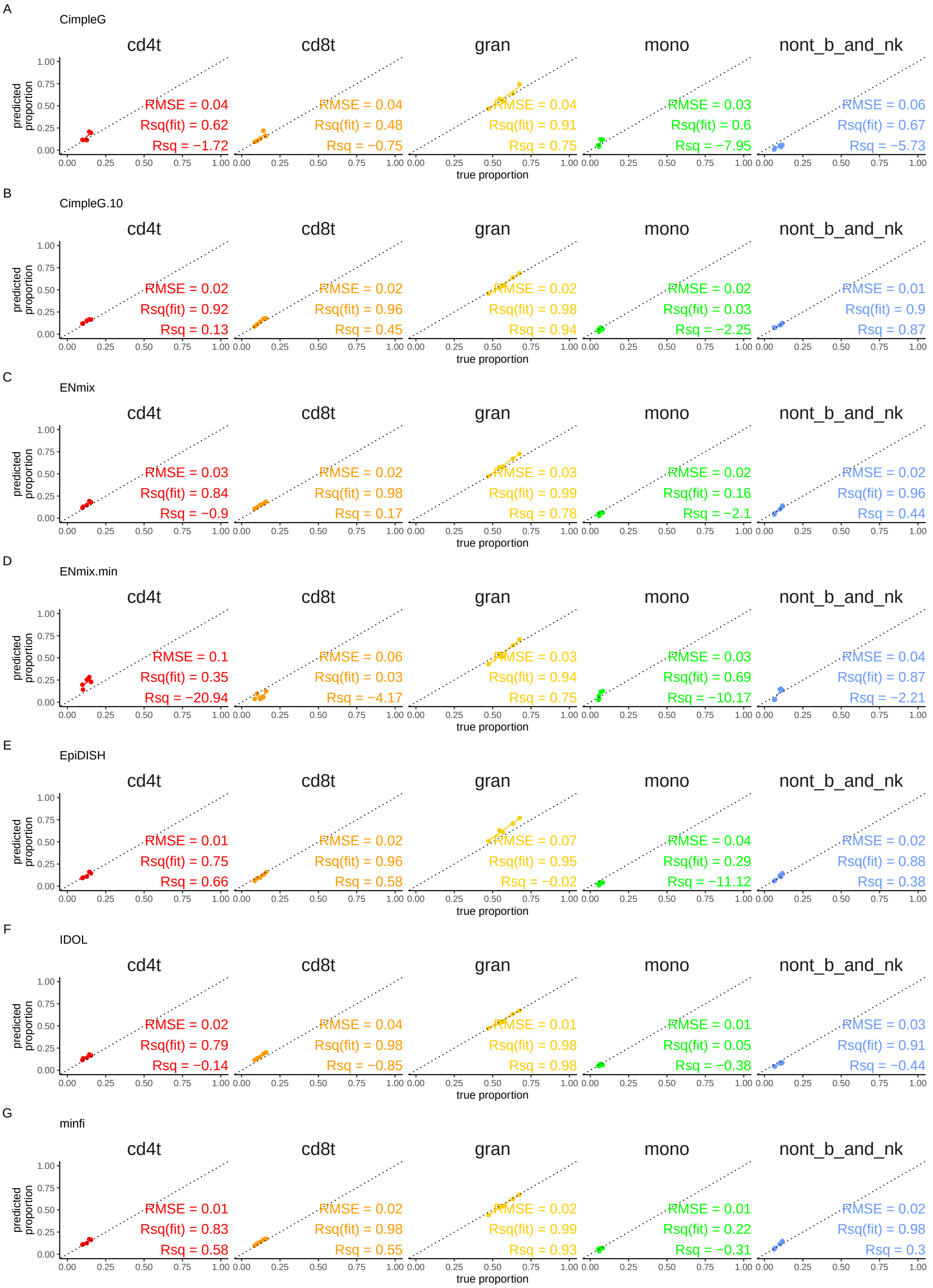

Fig. S9. The predicted deconvolution proportion (y-axis) vs real proportion (x-axis) for the Leukocyte dataset 3 for all deconvolution methods (continues in S10). Predictive accuracy statistics, RMSE, R^2 and R^2(fit), are shown for each method-target pair.

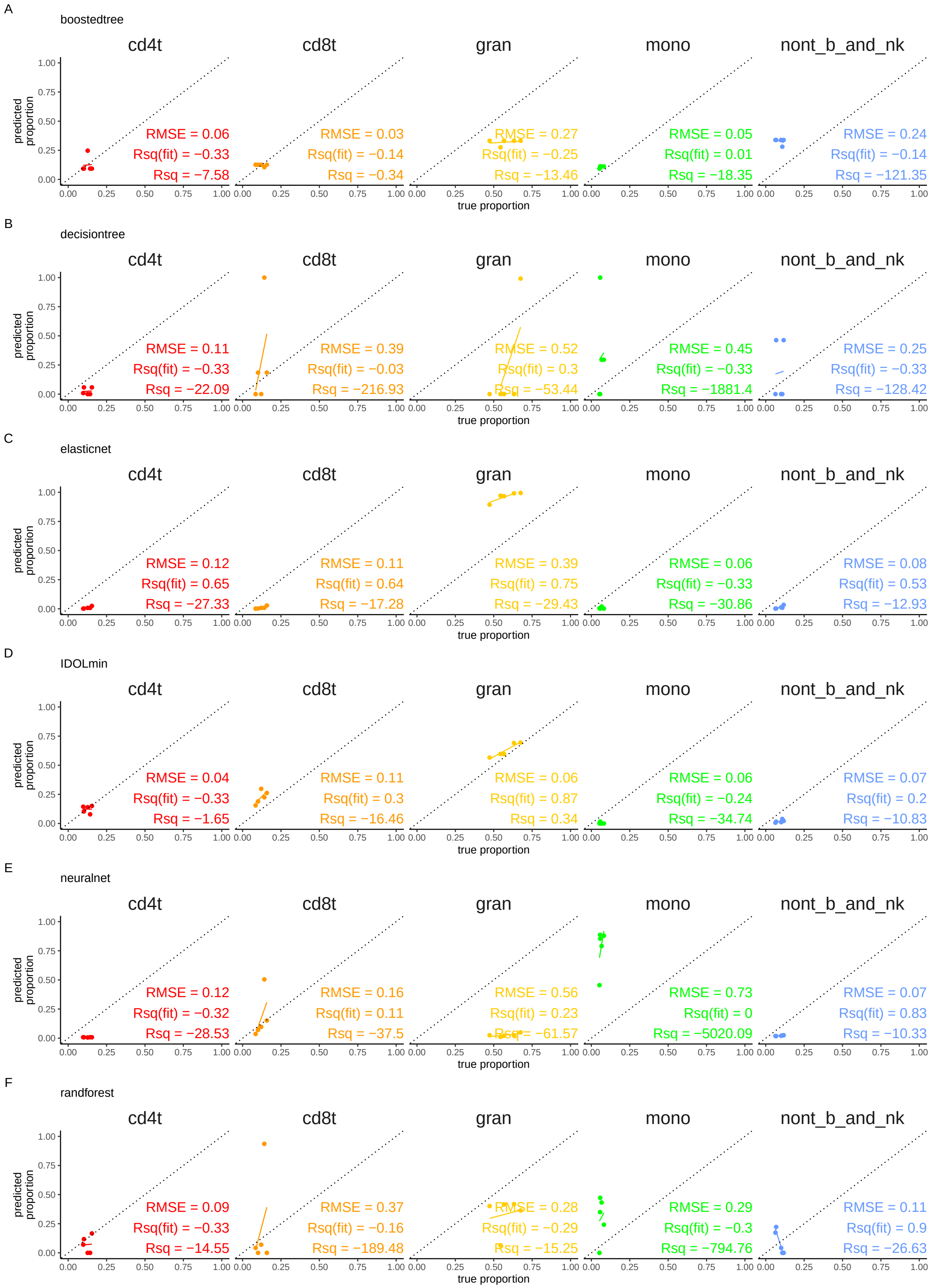

Fig. S10. The predicted deconvolution proportion (y-axis) vs real proportion (x-axis) for the Leukocyte dataset 3 for all deconvolution methods (continues from S9). Predictive accuracy statistics, RMSE, R^2 and R^2(fit), are shown for each method-target pair.

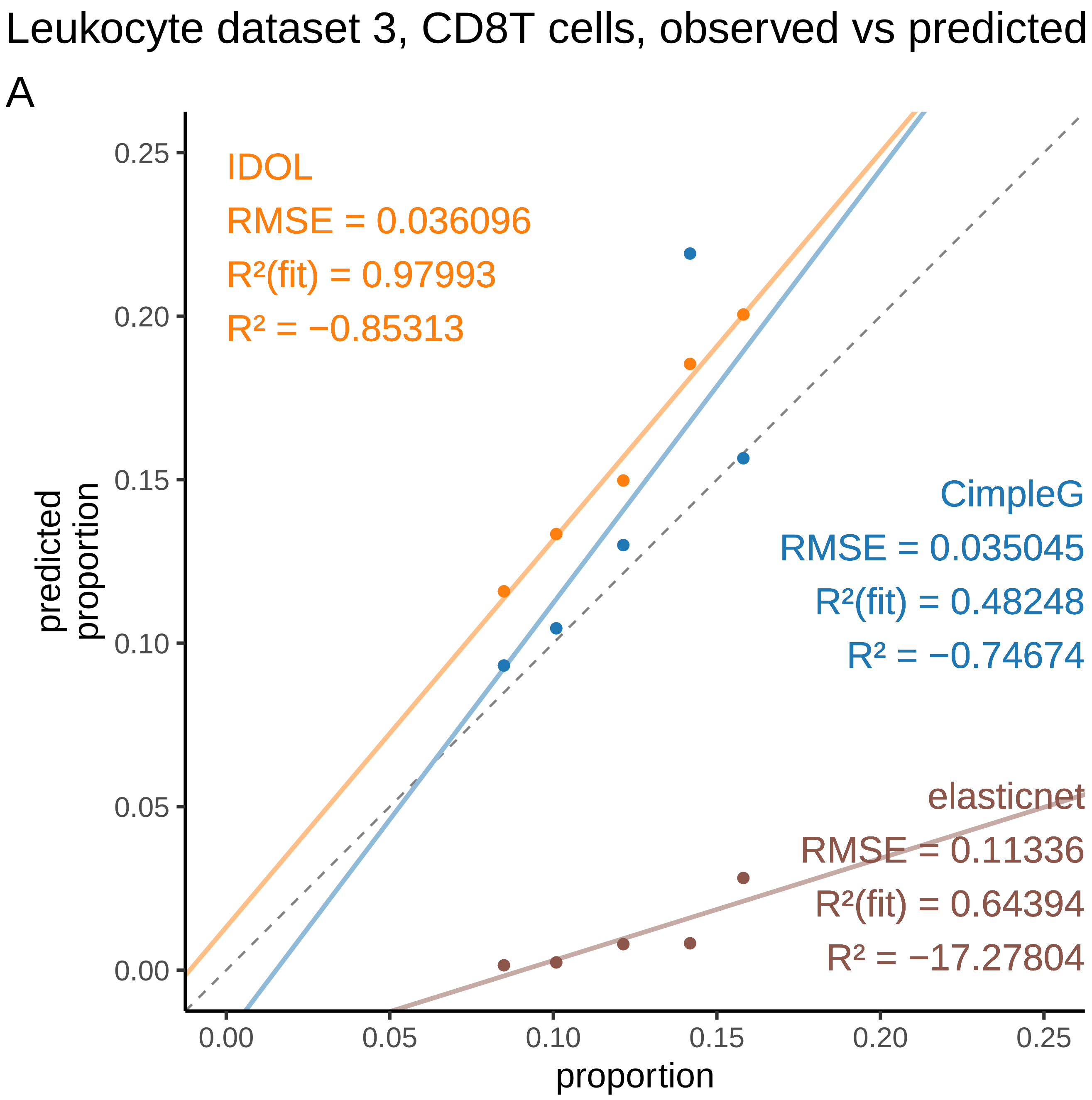

Fig. S11. Scatterplot with observed vs predicted deconvolution results scatterplot for the CD8T cells for the elasticnet, IDOL and CimpleG models. We observe that the R^2(fit) of IDOL is best (0.97) followed by Elastic Net (0.64) and CimpleG (0.48). However, IDOL has an slightly higher error (0.036) than CimpleG (0.035). It is clear from the plot that IDOL systematically overestimates the proportion of CD8 T cells and the R^2(fit) is not able to capture this. Another example is the Elasticnet, which has a higher R^2(fit) than CimpleG, while it drastically underestimates proportions, as reflected by its high error (0.11 RMSE). These results illustrate the fact the R^2(fit) only measures goodness-of-fit, but does not reflect the accuracy of the predictions.
