# Supplementary material for: CimpleG: finding simple CpG methylation signatures
Source: Genome Biol. 2023 Jul 10;24:161. doi: 10.1186/s13059-023-03000-0 (PMC10332104; doi:10.1186/s13059-023-03000-0)
Supplement: Supplementary file 3 — Additional file 3. Review history. [file 13059_2023_3000_MOESM3_ESM.docx]

**1^st^ round**

**Reviewer 1**

In this manuscript the authors present a method for identifying DNA methylation probes from Illumina methylation arrays for cell type deconvolution. Their method is to use a t-statistic to identify probes that separate one cell type from all others in the training data, and then evaluate the top probes using linear regression and AUPR to pick a single probe. They then compare this to several other classification methods (e.g. RandomForests) that have built in methods for feature selection. Their results show that selecting a single probe to identify each cell works fairly well for cell type deconvolution. In some ways this is an interesting study in that the idea that a single probe could be used for each cell type sounds absurd on its surface, but as they show, in practice this works surprisingly well. The argument is that by having such a reduced probe set, it would facilitate development of assays that probe this small set and enable clinical usage of such methods. The paper is well written, the methods well described, and I do find their argument about finding reductive probe sets to facilitate practical assay implementation in research or clinical settings to be compelling. My concern with this paper is that there are many other methods (e.g. EpiDish, IDOL, methods from Houseman et al. 2012) for determining a reduced probe-set that are not discussed in the intro or are not compared to in the presented manuscript. While typically these methods aren't pushed to identify such a small probe set, in theory for some of these one could push the parameters to select a small subset of probes about the same size as that used by CimpleG. At the end of the day, since they don't compare to these other methods, it is difficult to know how much accuracy is lost when going to such a small probe set and whether that loss in accuracy is biologically and /or practically meaningful. And without exploring this, I have trouble figuring out how I would design a simple assay for cell-type deconvolution. Because of this, I think it becomes hard to know how and if they are advancing the field. I also feel that without taking the step to develop an assay, which admittedly goes well beyond the current presented work, this is not of broad enough interest for Genome Biology. My feeling is that even if they included the analyses recommended below (which does not include designing and testing an assay), I'd still recommend that this manuscript is better suited for a more specialized journal.

Detailed Comments

IDOL is one of the better deconvolution methods out there, and they show in their papers that methylation state of an individual CpG can differentiate individual cell types for at least some types. IDOL, like CimpleG, uses a t-statistic for CpG selection, but then uses an iterative process to identify a user-defines subset of probes that minimizes deconvolution error. I didn't see mention of this past, similar work in their introduction.

Their initial 2016 paper also highlights how choosing good probe sets is critical for good deconvolution. In this respect I would expect the authors to mention that such methods have been employed in the past for probe selection in the background. I also thought the background was light on describing much of this prior work and instead focused on Elastic Net primarily.

It appears that IDOL (Koestler et al. 2016 BMC Bioinformatics, Salas et. al. Nat Comm 2022) achieves vastly superior results in terms of R2 and RMSE. Further, the latest version separates 12 cell types as opposed to the six used in Fig. 4 in this work. Obviously, the caveat is that it isn't on the same evaluation data, so the comparison may not be entirely fair, but it reinforces the need for comparing other methods and CimpleG in this work.

Given their interest in finding a reductive probe set, which I do think is an interesting idea, I think the authors need to compare what they are giving up versus well trained models with larger probe sets. In particular, I think it would be useful for the field if the authors explore this much deeper. For example, they could reduce the size of the probe set gradually selected by IDOL (or other methods) and see how the error increases as a function of probe set size to find the optimal size and compare that to their ultimate reduction of 1 CpG per cell type (e.g. while there is perfect separation in the probes in Fig. 3, the separation is not as good in probes/cell types in Fig. S4). This would likely give a better feel for the tradeoffs between how many CpGs you need to measure and how it affects the deconvolution accuracy.

As far as I can tell, all the bench marking was performed on artificial mixtures. The authors should include bench marking methylation data from available samples (i.e. "real data"), which have then been analyzed for cell-type composition using traditional FACS (e.g. GSE110530).

On p. 9 they discuss why using more features as selected by other methods performed worse and suggested this might be due to missing data. Whether missing data was the issue would be very easy to check -- and they should if they plan to make that claim. My guess though is that it has more to do with the size of the training data. In theory, if a single probe were much better at prediction, given enough training data these methods should be able to learn (e.g. by adjusting the weights) that the one value is the one to rely on and thus perform equally well.

When I ran the test example of the CimpleG from their GitHub instructions, I kept hitting errors about missing packages (list below). I was using a pretty fresh R installation. These package dependencies should be added to the install. Once I installed the packages, everything ran fine. I think it would be good to include some more details about the input data/files and the outputs in the instructions, but it was pretty intuitive from the test data.

Missing packages:

ggsci

patchwork

ggExtra

ggrepel

nnls

gtools

forcats

**Reviewer 2**

The manuscript by Maie et al entitled "CimpleG: Finding simple CpG methylation" is looking for alternatives using a simplified set of CpGs to estimate cell composition using DNA methylation. They compare decision trees, random forest neural network and their CimpleG method for selection of the CpGs used as input. The method improves the time processing and reduces the list of CpGs to 10 to 6 CpGs (one per cell type), aiming for its application in clinical settings. the method seems statistically sound, basing the selection in a modified AUC methods (AUPR). They used a large set of publicly available datasets, but some information about the testing and training are unclear and require additional information:

1. Tables 1 and 2 and supplementary material: The leukocyte sets include peripheral blood and umbilical cord blood from both healthy subjects and adults with diseases. This seems potentially problematic, as it has been shown that some of the cell subpopulations are different between newborns and adults, and the risk of cross contamination with other cell types may alter the signals. Could you elaborate about the rationale of why you decided to combine these samples? or how these potential technical issues were mitigated by your algorithm?

2. Table 1 and 2 and supplementary material: Similarly for the somatic subpopulations could you explain the sources (fetal or adult) for the different samples used in the algorithm?

3. Could you check the results in Figures 4? Why some r squared are negative and larger than 1? Is this a bug in your code or it is really that the result is so poorly fit that it is negative?

4. For Figure 4, could you elaborate on the RMSEs observed for individual cell types? In particular results for granulocytes, CD4T, CD8T, and NK cells seem quite large. Could you compared to other methods such as those of Jaffe, Koestler, EpiDISH, or Salas for comparison? this should be discussed in detail in your discussion section in particular if the intention is to translate these findings to the clinical setting.

**Authors’ response**

Reviewer #1

Reviewer #1: In this manuscript the authors present a method for identifying DNA methylation probes from Illumina methylation arrays for cell type deconvolution. Their method is to use a t-statistic to identify probes that separate one cell type from all others in the training data, and then evaluate the top probes using linear regression and AUPR to pick a single probe. They then compare this to several other classification methods (e.g. RandomForests) that have built in methods for feature selection. Their results show that selecting a single probe to identify each cell works fairly well for cell type deconvolution. In some ways this is an interesting study in that the idea that a single probe could be used for each cell type sounds absurd on its surface, but as they show, in practice this works surprisingly well. The argument is that by having such a reduced probe set, it would facilitate development of assays that probe this small set and enable clinical usage of such methods. The paper is well written, the methods well described, and I do find their argument about finding reductive probe sets to facilitate practical assay implementation in research or clinical settings to be compelling.

Authors’ Response: We thank the reviewer for the positive feedback regarding the relevance of the work.

Reviewer #1 comment: My concern with this paper is that there are many other methods (e.g. EpiDish, IDOL, methods from Houseman et al. 2012) for determining a reduced probe-set that are not discussed in the intro or are not compared to in the presented manuscript. While typically these methods aren't pushed to identify such a small probe set, in theory for some of these one could push the parameters to select a small subset of probes about the same size as that used by CimpleG. At the end of the day, since they don't compare to these other methods, it is difficult to know how much accuracy is lost when going to such a small probe set and whether that loss in accuracy is biologically and /or practically meaningful. And without exploring this, I have trouble figuring out how I would design a simple assay for cell-type deconvolution. Because of this, I think it becomes hard to know how and if they are advancing the field.

Authors’ Response: Indeed, our previous version of this work was focused on the cell- specific signature problem. We have expanded the manuscript to also cover the cell deconvolution problem by evaluating additional deconvolution methods and deconvolution data sets. See details below. We think this further clarifies the importance of CimpleG in finding small signatures for both problems: cell signature and cell deconvolution for a clinical setting.

Reviewer #1 comment: IDOL is one of the better deconvolution methods out there, and they show in their papers that methylation state of an individual CpG can differentiate individual cell types for at least some types. IDOL, like CimpleG, uses a t-statistic for CpG selection, but then uses an iterative process to identify a user-defines subset of probes that minimizes deconvolution error. I didn't see mention of this past, similar work in their introduction. Their initial 2016 paper also highlights how choosing good probe sets is critical for good deconvolution. In this respect I would expect the authors to mention that such methods have been employed in the past for probe selection in the background. I also thought the background was light on describing much of this prior work and instead focused on Elastic Net primarily.

Authors’ Response: We have now expanded the introduction to describe further approaches for feature selection used in cell deconvolution methods such as IDOL, Epidish, ENMix and the method described in Jaffe et al. (minfi). These are now found in the introduction and discussion (Section 1, Section 3).

Reviewer #1 comment: It appears that IDOL (Koestler et al. 2016 BMC Bioinformatics, Salas et. al. Nat Comm 2022) achieves vastly superior results in terms of R2 and RMSE. Further, the latest version separates 12 cell types as opposed to the six used in Fig. 4 in this work. Obviously, the caveat is that it isn't on the same evaluation data, so the comparison may not be entirely fair, but it reinforces the need for comparing other methods and CimpleG in this work.

Authors’ Response: To address this, we expand our deconvolution method evaluation by using additional data sets and additional methods (IDOL, EpiDISH, Minfi [Jaffe et al. 2014], ENmix). Our evaluation now includes 3 data sets: Leukocytes 1 (artificial mixture), Leukocytes 2 (artificial mixture) and Leukocytes 3 (real mixtures) all analysed in Salas et al 2022. Of note, our previous results were based on the now-named Leukocytes 1 data. Note also that we evaluate the Leukocyte 1 and 2 data sets with 12 specific cell types or with broader groups, covering 6 major cell types, as some methods can only be used for major leukocyte types (see below). Next, it is also important to clarify that the R2 definitions from our work and IDOL publications are distinct. IDOL papers (and others) calculate the R2 on the fitted model (fitted R2), while we estimate the R2 assuming the diagonal line as the ideal result (diagonal R2). Furthermore, in both IDOL manuscripts, the R2 fit is used for optimization, while results are evaluated with the Pearson R2 statistic. In our understanding having a good fit R2 (all predictions lie in the estimated line) does not mean that the predictions are accurate, as the line can under/over-estimate the proportions systematically. The diagonal fit actually shows how much the predictions deviate from the correct estimates. We include an example of this and all results with both R2 estimates in the supplement (Sup. Fig. 9-11) and include a discussion about this topic in the discussion section (Section 3). In short, we argue that the fitted R2 estimates are misleading and the RMSE (root mean squared error) should be the major statistic used for benchmarking cell deconvolution methods. Regarding deconvolution workflows, it is important to highlight that some of these methods can be seen as stand-alone workflows (such as IDOL and CimpleG), as they perform both signature detection and deconvolution in an integrated manner, while other methods, such as ENmix and minfi depend on previously generated deconvolution reference datasets. We evaluated ENmix, EpiDISH and minfi using their signatures, which only supported 6 major leukocyte types. Therefore, they could not be evaluated when using 12 leukocyte signatures.

In short, our cell deconvolution benchmarking indicates that IDOL, a version of CimpleG with 10 CpG (CimpleG.10) per cell type and minfi are overall the top performers (Fig. 4 and Sup. Fig. 6-8). We have also evaluated a version of IDOL and ENmix by using the minimum possible number (1 or 2) CpGs per cell type. We observe that CimpleG outperforms all single signature methods, while having competitive RMSEs in relation to the top-performing methods (see Section 2.3). Altogether these results reinforce the discriminative power of small CpG signatures in the cell deconvolution problem.

Reviewer #1 comment: Given their interest in finding a reductive probe set, which I do think is an interesting idea, I think the authors need to compare what they are giving up versus well trained models with larger probe sets. In particular, I think it would be useful for the field if the authors explore this much deeper. For example, they could reduce the size of the probe set gradually selected by IDOL (or other methods) and see how the error increases as a function of probe set size to find the optimal size and compare that to their ultimate reduction of 1 CpG per cell type (e.g. while there is perfect separation in the probes in Fig. 3, the separation is not as good in probes/cell types in Fig. S4). This would likely give a better feel for the tradeoffs between how many CpGs you need to measure and how it affects the deconvolution accuracy.

Authors’ Response: This is an interesting point. We now evaluate IDOL with distinct signature sizes (default parameters vs. the minimum signature possible; 1 per cell type + 1). The same was performed for ENmix, where two CpGs per cell type is possible. On the other hand, we also now include a version of CimpleG with 10 CpG sites per cell type to evaluate how the method performs with larger signatures. Our benchmarking results indicate that CimpleG (one site per cell) outperforms all other small signature methods in the 3 evaluated data sets (see Fig. 4, and Sup. Fig. 6-8) Regarding the 10 CpG signature version of CimpleG, it had a competitive performance against the top performing IDOL and minfi.

Reviewer #1 comment: As far as I can tell, all the benchmarking was performed on artificial mixtures. The authors should include benchmarking methylation data from available samples (i.e. "real data"), which have then been analyzed for cell-type composition using traditional FACS (e.g. GSE110530).

Authors’ Response: We thank the reviewer for this remark. We now also include this real cell mixture data in our cell deconvolution benchmarking, as Leukocyte dataset 3. Here, minfi was the best-performing method, followed by CimpleG (10 CpGs) and ENmix. CimpleG with one CpG had the lowest RMSE when compared to other small signature methods and had a performance as good as the top performing method according to the (Friedman and Nemenyi post-hoc test; Fig. 4).

Reviewer #1 comment: On p. 9 they discuss why using more features as selected by other methods performed worse and suggested this might be due to missing data. Whether missing data was the issue would be very easy to check -- and they should if they plan to make that claim. My guess though is that it has more to do with the size of the training data. In theory, if a single probe were much better at prediction, given enough training data these methods should be able to learn (e.g. by adjusting the weights) that the one value is the one to rely on and thus perform equally well.

Authors’ Response: Indeed, the text regarding missing data was misleading/unclear. In short, DNAm array pre-processing pipelines can indicate a missing value, when probe signal value for a particular array is of low quality. As this is a site-specific problem, the larger the data, the larger is the chance that this happens. Note that the evaluated methods can not cope with missing data and this is indeed a technical problem. An alternative to this problem, the use of imputation, is rarely explored, but it is now supported by CimpleG. We change the discussion to indicate that this is currently an unaddressed issue in the field, but not an explanation for the results (see Section 3).

Reviewer #1 comment: When I ran the test example of the CimpleG from their GitHub instructions, I kept hitting errors about missing packages (list below). I was using a pretty fresh R installation. These package dependencies should be added to the install. Once I installed the packages, everything ran fine. I think it would be good to include some more details about the input data/files and the outputs in the instructions, but it was pretty intuitive from the test data.

Missing packages:

ggsci

patchwork

ggExtra

ggrepel

nnls

gtools

Forcats

Authors’ Response: We apologise for the issues. We have now changed the installation instructions to be as comprehensive as possible.

Reviewer #2

Reviewer #2: The manuscript by Maie et al entitled "CimpleG: Finding simple CpG methylation" is looking for alternatives using a simplified set of CpGs to estimate cell composition using DNA methylation. They compare decision trees, random forest, neural network and their CimpleG method for selection of the CpGs used as input. The method improves the time processing and reduces the list of CpGs to 10 to 6 CpGs (one per cell type), aiming for its application in clinical settings. the method seems statistically sound, basing the selection in a modified AUC methods (AUPR). They used a large set of publicly available datasets, but some information about the testing and training are unclear and require additional information:

Authors’ Response: We thank the reviewer for the positive comments regarding the importance and the statistical robustness of our work.

Reviewer #2 comment 1: Tables 1 and 2 and supplementary material: The leukocyte sets include peripheral blood and umbilical cord blood from both healthy subjects and adults with diseases. This seems potentially problematic, as it has been shown that some of the cell subpopulations are different between newborns and adults, and the risk of cross contamination with other cell types may alter the signals. Could you elaborate about the rationale of why you decided to combine these samples? or how these potential technical issues were mitigated by your algorithm?

Authors’ Response: Our sample selection has as its primary objective to be as inclusive as possible to maximise sample size and include biological variability, this in turn increases our signature selection power. We included some disease samples, such as some CMV seropositive (citomegalovirus) NK cells and some monocytes with multiple sclerosis. Both cell types also include some coord blood/fetal cells, which were only used in the test data set. To double check if these biological covariates affect DNAm signature, we now show in Sup. Figure 5, how DNAm values are related to the source/sample covariate status. We observe that disease or fetal vs. adult status did not affect the DNAm values of CimpleG selected CpG sites. This is now discussed on page 7.

Reviewer #2 comment 2: Table 1 and 2 and supplementary material: Similarly for the somatic subpopulations could you explain the sources (fetal or adult) for the different samples used in the algorithm?

Authors’ Response: We now include relevant covariate information (fetal vs adult and healthy vs. disease) in the new Supplementary Tables 2 and 3, as requested.

Reviewer #2 comment 3: Could you check the results in Figures 4? Why some r squared are negative and larger than 1? Is this a bug in your code or it is really that the result is so poorly fit that it is negative?

Authors’ Response: Indeed, the negative values are associated with poor fits, which are particularly the case for some methods (Random Forests). These are now shown in Fig. S9-S10 of the current manuscript. Note that our original R2 definition is distinct from the one used in other works, such as IDOL (Salas et al. 2022). There, they calculate the R2 on the model fitted line (fitted R2), while we use R2 on the diagonal line (diagonal R2). We use this definition (diagonal R2), because the diagonal line should represent the ground truth. Indeed, with this definition, some methods do quite badly. We include in the manuscript results with both R2 versions and a discussion about this (see Section 3 and Sup. Fig. 9-11). We think this is an overlooked, but important aspect in the analysis of cell mixtures, which leads to confusion in the field. We argue in the manuscript that to avoid confusion, the RMSE should be the only statistic used for the evaluation of cell deconvolution prediction results.

Reviewer #2 comment 4: For Figure 4, could you elaborate on the RMSEs observed for individual cell types? In particular results for granulocytes, CD4T, CD8T, and NK cells seem quite large. Could you compare to other methods such as those of Jaffe, Koestler, EpiDISH, or Salas? this should be discussed in detail in your discussion section in particular if the intention is to translate these findings to the clinical setting.

Authors’ Response: To make a fair comparison with competing methods, we expanded our benchmarking to evaluate EpiDiSH, IDOL (Koestler et al. 2016, Salas et al. 2022) and Jaffe et al. (minfi) and ENmix. We also include two additional deconvolution data sets from Salas et al. 2022 (now denoted Leukocyte 2 and Leukocyte 3). As requested by reviewer 1, we also evaluated variants of methods (IDOL, ENMix and CimpleG) with the use of large (default setting) or minimal signatures. For some data sets (Leukocytes 1 and 2), the evaluation was performed either on 12 or 6 cell types, as some approaches could only be executed with 6 major leukocytes. We observe that IDOL and CimpleG with 10 CpGs per cell type are the best overall methods. Also, CimpleG with small signatures outperforms all methods based on small signatures and has a RMSE equivalent to the top-performing methods.

Authors’ Response: Of note, the large RMSEs were associated with poor signatures from methods like decision trees, random forests, boosted trees and neural networks (see Fig. S9-S10). An explanation is the fact that these signatures were obtained in the problem of cell type prediction but not cell deconvolution; these are not specifically trained for deconvolution, but for classification. We do not find such large RMSEs in top-performing cell deconvolution methods (CimpleG, ENMix, EpiDISH IDOL, minfi and Elastic Net) in novel and expanded analysis. These results are now integrated into the main manuscript (Section 2.3 and Fig 4 and Sup. Fig. 6-10). In the discussion, we now also include a text regarding the importance of the proposed signatures in a clinical context (Section 3).

**2^nd^ round**

**Reviewer 1**

I appreciate the work the authors invested to further benchmarking CimplG against other methods. I realize that was no small endeavor, but I do think it improves the paper substantially and highlights to me what is still a surprising but interesting finding about how few CpGs are needed to distinguish different cell types. I only had a couple minor comments about passages in the text:

Minor Comments:

Page 11: The sentences about why methylation has not been used in the clinic more needs to be changed. I agree reducing probes is valuable, but probe set size is not the reason methylation diagnostics are not in the clinic. More likely this has to do with quality of the analysis and limitations/difficulties of bisulfite-based chemistries. Some examples: PAM50 was a 50 gene panel that was clinically used. Nanostring nCoutner is CLIA certified and 800 probes (RNA or DNA). Clinical exome sequencing is >50 million bp of data, but it’s used. WGS of fetal DNA in the mother’s blood is now a standard way to look for trisomies and genetic anomalies in the feturs. All of these range from about the same size to much larger in terms of the number of sites examined for the assay, and yet all are used clinically. Genome-wide analysis is challenging, but totally possible if valuable enough. And to samples 100s or even 1000s of sites is possible, if warranted clinically. Obviously, though I agree with the statement that smaller probe set sizes will be cheaper and easier to implement.

Page 5: While I do think that reporting the algorithm speeds is important and useful, I think the language around how important this is needs to be changed. I’m going to guess that by using parallelization, the one probe CimpleG could be sped up tremendously. I don’t think the authors should do this, but they should mention it’s possible. Also, the speeds are the training speeds (for deconvolution or signature generation), not the diagnostic/classification speed. It will take much more than 6 hours (which is slowest algorithm) to assemble hundreds of data sets used for training. I.e. if you include gathering data, preprocessing, and then generating signature, the final signature generation time is pretty small and will not really affect the length of time to go from nothing to signature. The classification speed for each of these should be very fast. I think most people in the field will know this, but given the broader audience of the journal these statements about speed need to be properly put in context.

**Reviewer 2**

The manuscript by Maie et al entitled "CimpleG: Finding simple CpG methylation" is looking for alternatives using a simplified set of CpGs to estimate cell composition using DNA methylation. They compare decision trees, random forest,

neural network and their CimpleG method for selection of the CpGs used as input. The method improves the time processing and reduces the list of CpGs to 10 to 6 CpGs (one per cell type), aiming for its application in clinical settings. the method seems statistically sound, basing the selection in a modified AUC methods (AUPR).

The authors provided enough information about the different methodologies and how their methods compares to current developments, as well as the potential limitations of their approach.

Minor comment:

As you used SeSaMe for preprocessing, please include the version of the library used and the order in which the steps were performed. This is critical for reproducibility with this specific library. Please include versions of the other libraries used as needed.

**Authors’ response**

Reviewer #1 Minor Comments:

Page 11: The sentences about why methylation has not been used in the clinic more needs to be changed. I agree reducing probes is valuable, but probe set size is not the reason methylation diagnostics are not in the clinic. More likely this has to do with quality of the analysis and limitations/difficulties of bisulfite-based chemistries. Some examples: PAM50 was a 50 gene panel that was clinically used. Nanostring nCoutner is CLIA certified and 800 probes (RNA or DNA). Clinical exome sequencing is >50 million bp of data, but it’s used. WGS of fetal DNA in the mother’s blood is now a standard way to look for trisomies and genetic anomalies in the feturs. All of these range from about the same size to much larger in terms of the number of sites examined for the assay, and yet all are used clinically. Genome-wide analysis is challenging, but totally possible if valuable enough. And to samples 100s or even 1000s of sites is possible, if warranted clinically. Obviously, though I agree with the statement that smaller probe set sizes will be cheaper and easier to implement.

Answer: We understand the critical viewpoint of the referee. We added the following sentence in the discussion to reflect this. “In principle, epigenetic diagnostics with genome-wide analysis is feasible – deep sequencing technology is also used for screening of mutations and chromosomal abnormalities in the clinics - however, the accreditation process is more challenging, as compared to targeted assays. For example, the Illumina BeadChip microarray platforms are so far not accredited for clinical diagnostics.”

Page 5: While I do think that reporting the algorithm speeds is important and useful, I think the language around how important this is needs to be changed. I’m going to guess that by using parallelization, the one probe CimpleG could be sped up tremendously. I don’t think the authors should do this, but they should mention it’s possible. Also, the speeds are the training speeds (for deconvolution or signature generation), not the diagnostic/classification speed. It will take much more than 6 hours (which is slowest algorithm) to assemble hundreds of data sets used for training. I.e. if you include gathering data, preprocessing, and then generating signature, the final signature generation time is pretty small and will not really affect the length of time to go from nothing to signature. The classification speed for each of these should be very fast. I think most people in the field will know this, but given the broader audience of the journal these statements about speed need to be properly put in context.

Answer: We agree with the reviewer that the great majority of the algorithms require less computing time than low-level analysis of DNA methylation. We have added the following sentence in the results section to reflect this “Of note, the computing time of the algorithms presented here does not represent a crucial aspect of the analysis. This is because the computing times are much lower than the time necessary for measuring or pre-processing the DNA methylation data.”.

Reviewer #2

Minor comment: As you used SeSaMe for preprocessing, please include the version of the library used and the order in which the steps were performed. This is critical for reproducibility with this specific library. Please include versions of the other libraries used as needed.

Answer: The exact version (sesame R package v1.16.1) as well as the pre-processing argument specifying the steps performed (“QGCDPB”) are now explicit in the manuscript. We also provide the code used for the analysis at https://doi.org/10.5281/zenodo.8045462.
